# Localization of Sesquiterpene Lactones Biosynthesis in Flowers of *Arnica* Taxa

**DOI:** 10.3390/molecules28114379

**Published:** 2023-05-27

**Authors:** Agata Parafiniuk, Krystyna Kromer, Mariusz G. Fleszar, Agnieszka Kreitschitz, Jerzy Wiśniewski, Andrzej Gamian

**Affiliations:** 1 Laboratory of Tissue Cultures, Botanical Garden, Faculty of Biological Sciences, University of Wroclaw, Sienkiewicza 23, 50-525 Wroclaw, Poland; 2 Department of Biochemistry and Immunochemistry, Wroclaw Medical University, Chalubinskiego 10, 50-368 Wroclaw, Poland; mariusz.fleszar@umw.edu.pl; 3 Department of Plant Development Biology, Faculty of Biological Sciences, University of Wroclaw, ul. Kanonia 6/8, 50-328 Wroclaw, Poland; agnieszka.kreitschitz@uwr.edu.pl; 4 Department of Biochemistry, Molecular Biology and Biotechnology, Faculty of Chemistry, Wroclaw University of Science and Technology, 50-370 Wroclaw, Poland; jerzy.wisniewski@pwr.edu.pl; 5 Hirszfeld Institute of Immunology and Experimental Therapy, Polish Academy of Sciences, Weigla 12, 53-114 Wroclaw, Poland; andrzej.gamian@hirszfeld.pl

**Keywords:** *Arnica montana*, *A. chamissonis*, sesquiterpene lactones, helenalin and dihydrohelenalin esters, biosynthesis localization, histochemical study

## Abstract

*Arnica montana* is a valuable plant with high demand on the pharmaceutical and cosmetic market due to the presence of helenalin (H) and 11α, 13-dihydrohelenalin (DH) sesquiterpene lactones (SLs), with many applications and anti-inflammatory, anti-tumor, analgesic and other properties. Despite the great importance of these compounds for the protection of the plant and their medicinal value, the content of these lactones and the profile of the compounds present within individual elements of florets and flower heads have not been studied so far, and attempts to localize these compounds in flower tissues have also not been conducted. The three studied *Arnica* taxa synthesize SLs only in the aerial parts of plants, and the highest content of these substances was found in *A. montana* cv. Arbo; it was lower in wild species, and a very small amount of H was produced by *A. chamissonis*. Analysis of dissected fragments of whole inflorescences revealed a specific distribution pattern of these compounds. The lactones content in single florets increased from the top of the corolla to the ovary, with the pappus calyx being a significant source of their production. Histochemical tests for terpenes and methylene ketones indicated the colocalization of lactones with inulin vacuoles.

## 1. Introduction

*Arnica montana* L., which belongs to the Asteraceae family, is a wild species found in the mountainous regions of central and southern Europe as well as on the plains of the Scandinavian countries. In all countries, with exception of Spain and Romania, it is a protected species. Due to the unavailability of raw materials with complex chemical compositions and wide cosmetic and pharmaceutical applications, arnica is a plant in increasing demand. Instead of *A. montana*, *A*. *chamissonis* subsp. *foliosa* is proposed, as it also contains helenalin and 11α, 13-dihydrohelenalin, as well as their oxy- and hydroxyl derivatives, such as arnifolins and chamissonolides. Mexican *Hetheroteca inuloides* Cass., containing cadinane sesquiterpenes, is also used as a substitute [1].

*Arnica montana* flowers contain terpenoids, including sesquiterpene lactones, diterpenes, triterpenes (arnidiol, faradiol), flavonoids, essential oils, phenolic acids (caffeic and chlorogenic, and their depsides), coumarins, tannins, saponins and phytosterols [2,3]. The most important secondary metabolites that are responsible for the properties of arnica belong to the biologically active SLs, organic compounds derived from terpenes. SLs are colorless lipophilic substances built with 15 carbon skeletons. These secondary metabolites are biosynthesized in plants from three molecules of farnesyl pyrophosphate (FPP), which is a product of the condensation reaction of one molecule of dimethylallyl pyrophosphate (DMAPP) and two molecules of isopentenyl diphosphate (PPI) catalyzed by farnesyl diphosphate synthase (FDS). PPI and DMAPP are synthesized in two independent metabolic processes, the mevalonate (MVA) and the 2-C-methyl-D-erythritol-4-phosphate (MEP) pathways, which are localized in cytosol and chloroplasts, respectively [4]. The first step in the biosynthesis of SLs is FPP cyclization catalyzed by sesquiterpene synthases (STPSs), resulting in the formation of γ-lactone, a precursor of lactones found in the Asteraceae family. STPSs are mainly localized in the cytosol and are characterized by plasticity, demonstrating the ability to reuse the substrate [5,6]. The cyclization reaction is followed by a series of oxidations and hydroxylations carried out by cytochrome P450 enzymes. Then, the structure of C15 can be modified by other enzymes, such as alcohol dehydrogenases, reductases, and acyl transferases. The diversity of STPSs and cytochrome P450s (CYPs) and their broad enzymatic activity results in a great diversity of SL structures [7]. The biogenetic scheme for SLs was proposed first by Herz in 1977 [8], and a few years later, Seaman arranged the different types of carbon skeletons into four columns that determined the successive stages of biogenesis. SLs present in arnica flowers belong to the group of pseudoguayanolides [9].

The chemical structure of the most of the natural SLs is complex, so their laboratory production generally involves multistep synthesis [10]. For the flagship H compound, a few different strategies of organic synthesis were proposed [11,12,13] with a small overall product yield, especially if the stereochemistry of a naturally occurring compound is of interest (for example H 6.6% [11]). Therefore, the research effort to increase SLs biosynthesis in plants is even more important.

Numerous SLs found in the plant, not just in Asteraceae, accumulate in specialized tissues, such as glandular trichomes, oil bodies, and resin channels, as well as in organs, e.g., in flowers, leaves, others in roots or fruits, and even in the whole plant [14]. The exact place of synthesis in cells, tissues, or plant organs has not yet been determined. Most likely, it is characteristic of the species. Recognition of the place of lactone biosynthesis is of fundamental importance for determining the physiological function of these compounds and their role in the life of the plant, but it may also facilitate the isolation of enzymes related to the biosynthesis of these compounds. Information regarding the presence of genes involved in the biosynthesis and accumulation of these compounds is valuable. This knowledge may prove also important in metabolic engineering efforts to increase the biosynthesis of SLs because pathway redundancy and involvement of multiple intracellular compartments [15] make genetic transformation difficult. Manipulation of a single enzymatic step often brings unpredictable effects and sometimes leads to defective plants, such as in tomato transgenic plants with overexpression of phytoene synthase [16,17]. A positive example of genetic modification and elimination of unwanted components in peppermint was the introduction of the antisense version of (+)-menthofuran synthase (+) and the introduction of elite transgenic lines [18]. Otherwise, the production of artemisinin in tobacco plants was achieved by introducing two mega-biosynthetic pathways separately into the chloroplast and nuclear genome [19].

**Figure 1:**
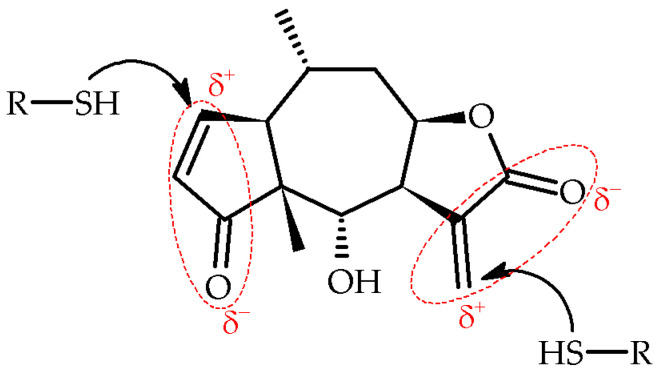
Reactive helenalin groups α,β-unsaturated carbonyl groups, from the left: cyclopentenone and α- methylene-γ-butyrolactones.

SLs present in the studied taxa, *Arnica montana* L., *A. montana* cv. Arbo, and *Arnica chamissonis* Less., are mainly H esters (helenanolides) and DH esters (dihydrohelenanolides) with carboxylic acids [3]. The anti-inflammatory activity of these compounds has been demonstrated in cellular immune responses to infections, stress, free radicals, UV radiation, and other factors. Lyss et al. proved that H and DH inhibit the activity of the nuclear factor kappa-light-chain-enhancer (NF-κB) of activated B cells, T cells, and epithelial cells in response to four different stimuli, eliminating gene expression controlled by NF-κB [20]. Hall et al. proved that the activity of these compounds is conditioned by the presence of an α-methylene-γ-lactone group and a hydroxyl group at C-6 carbon (Figure 1) [21].

Esterification or elimination of the C-6 hydroxyl group significantly reduces the anti-inflammatory activity of H. In addition, the presence of a ring of β-unsaturated cyclopentenone and α-epoxy-methylenecyclopentenone also determines the anti-inflammatory effect of the compound [21]; however, Jacobs et al. showed that helenalin acetate (HA) is a stronger inhibitor of CCAAT/enhancer binding protein (C/EBPβ) than NF-κB. HA is the first highly active low-molecular inhibitor of transcription factor C/EBPβ that inhibits C/EBPβ through a direct binding mechanism [22]. Both H and HA contain two α,β-unsaturated carbonyl groups (Figure 1).

These electrophilic moieties are known to undergo Michael addition reactions with nucleophiles, such as thiol cysteine residues [20,23]. Both compounds inhibit the activity of DNA binding to the transcription factor NF-κB by covalently modifying the Cys38 at the p65 subunit of NF-κB [20,24]. Jakobs et al. have shown that the activity of the lactones may depend on both reactive groups. To prove this, they investigated the inhibitory potential of two similar SLs, chamissonolid and 11α, 13-dihydrohelenalin acetate, both having only one of the reactive groups of HA in their structure. It turned out that these compounds were practically inactive compared to HA. The above studies confirmed that the specific spatial structure of these molecules, and not just the mere presence of two reactive groups, determine inhibitory activity [22]. On the other hand, studies have revealed that C/EBPβ is the regulatory link between granulocyte macrophage-colony stimulating factor (GM-CSF) signaling pathways and that C/EBPβ-deficient macrophages exhibit serious impaired proliferation of these cells [25], in which this complex stimulates fatty acid absorption and fat cell differentiation, and defective lipid metabolism is the primary cause of lung disease (PAP) and atherosclerosis.

The dried flower heads (*Arnicae anthodium*) containing not less than 0.4% of the sum of SLs expressed as dihydrohelenalin tigliniate (C_20_H_26_O_5_) are the pharmacopeial raw material [26]. Since *Arnica montana* is an endangered plant and its harvest from natural sites is impossible, the pharmacopeial raw material is obtained from Romania, Spain, where the species is not protected, and from the plantations [27,28,29,30].

Analyses of the production of *A. montana* under cultivation conditions suggested higher production in controlled crops compared to natural populations, preserving their content of SLs [27,28,29,31,32,33]. Such differences in SLs content may depend on genetic factors and abiotic conditions of the site, including altitude, temperature, and rainfall [28,31,34,35]. The literature data on the concentration and chemical composition of SLs in flowers come from research on either natural conditions or controlled plantations in Europe were presented as supplementary material (Table S1).

The location and spatial distribution of SLs biosynthetic sites in various plant species is important because of the unique properties of these metabolites and the potential for molecular manipulation to improve the yield of these substances. Trials and procedures were carried out to ascertain the sites of biosynthesis, including the immersion of organs in solvent solutions and the dissection and division of secretory glands, followed by the examination of extracts using high-performance liquid chromatography (HPLC) [36,37]. Identifying the active sites of the enzymes involved in SLs synthesis is an alternative method for carrying out these analyses. Specific germacrene A synthases have been found in *Helianthus annus* as germacrene A monooxygenase, which is expressed in stalk cells and the head of capitate GT secretory cells when a polyclonal antibody against HaGAO is fluorescently labeled [38]. The metabolic profile of *Smallanthus sonchifolius* and three lactone synthases responsible for its production were identified in a subsequent study by Lopes et al. [39]. Using photomicroscopic examinations and matrix-assisted laser desorption/ionization-mass spectrometry imaging (MALDI-MS), the aforementioned authors were able to identify the locations of their synthesis and accumulation simultaneously, confirming that glandular trichomes accumulate SLs. The viability of MALDI-MS imaging for the analysis of proteins and secondary metabolites within sunflower trichomes was confirmed by Silva et al., who emphasized the significance of optimizing parameters for such detections, challenges presented by trichome size, and cracking of glands during tissue dehydration [40]. In our study, we employed histochemical techniques that, when combined with the results of ultra-high performance liquid chromatography-mass spectrometry (UHPLC-MS) analysis, revealed the spatial distribution of SLs in *Arnica montana* flower heads.

This study aimed to identify and understand the localization of SLs biosynthesis in *Arnica* taxa, examine changes in synthesis ability, and determine the profile of these compounds in certain flower elements in subsequent stages of plant development. This study was supplemented by the subcellular localization of SLs in flower tissues based on histochemical tests.

## 2. Results

**Figure 2:**
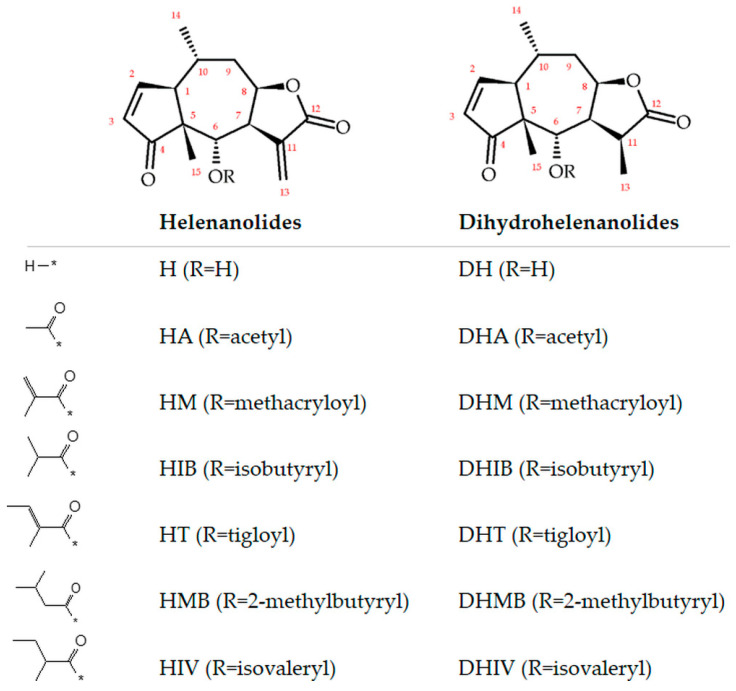
Examined derivatives of helenanolides (H) and dihydrohelenanolides (DH).

We have investigated the content of SLs, H and DH and their esters with acetic, isobutyric, methacrylic, tiglic, and other carboxylic acids (Figure 2).

### 2.1. SL Accumulation in Flower Heads in Arnica Taxa

The aerial parts of the plants were rich in SLs. All of the studied SLs were present in flowers. The highest content of SLs was found in flowers in the full flowering stage in *A. montana* cv. Arbo (24.88 mg/g dw, od dry weight), medium content of lactones was in *A. montana* (11.63 mg/g dw), and the lowest content in *A. chamissonis* (0.14 mg/g dw) (Table 1). The composition of lactone derivatives occurring in flowers within the same species *Arnica* was similar but different between individual species. *A. chamissonis* synthesizes another type of lactones of the pseudoguayanolide type, including chamissonolides and arnifolins and their oxy- and hydroxyl derivatives; therefore, the spectrum of the compounds present was much poorer than in *A. montana* in terms of H and DH and their esters. *A. chamissonis* was characterized by the presence of only H, interestingly especially at the beginning of flowering (in buds) (0.24 mg/g dw).

In *Arnica montana* taxa, as the flowers matured (from the bud to the overblown inflorescence), the lactone content increased in both representatives by about 2.5 times. The opposite trend in the studied compounds was observed in *A. chamissonis* flowers, in which the H content decreased at the full flowering stage from 0.26 mg/g dw to 0.14 mg/g dw.

In the flowers of both *Arnica montana* taxa (Table 1)*,* H dominated over DH, and the proportions between these groups of compounds changed during flowering. At the beginning of flowering (buds) in *A. montana*, the ratio between the two groups of derivatives (H/DH) was 1:2, but as the flowers were maturing, this ratio equalized to 1:0.9. In *A. montana* cv. Arbo, the predominance of H was already noted as flowers starting flowering 1.4:1, and during flowering, this ratio increased two times (2.9:1). The correlations of the two studied groups of SL derivatives in the leaves (Table S2) looked different than in flowers, where DH dominated over H. As was in flowers, the highest content of SLs was found in the leaves of the *A. montana* cv. Arbo variety (total 7.55 mg/g dw), almost 8 times less in *A. montana* (total 0.98 mg/g dw), and the lowest content was found in *A. chamissonis* (total 0.54 mg/g dw).

### 2.2. SLs in Leaves

In the leaves (Table S2), the composition of lactones significantly differs from that found in flowers; namely, there is a reversal of the relations between the derivatives of both forms of lactones. In these organs a significant predominance of DH esters over H esters was noted, where there are almost five times more of them in the *A. montana* cv. Arbo. On the other hand, a wild species from Lower Silesia, does not contain H esters at all, while the leaves of *A. chamissonis* are characterized by the presence of a small amount of H (0.16 mg/g dw). In both *A. montana* and *A. montana* cv. Arbo, the dominant metabolites in leaves were methacryloyldihydrohelenalin (DHM) (*m/z* 333.17), tigloyldihydrohelenalin (DHT) (*m/z* 347.19), 2-methylbutyryldihydrohelenalin (DHMB), and isovaleryldihydrohelenalin (DHIV) (*m/z* 349.20). In the underground parts of the plants, i.e., the rhizome and roots, SLs were not found or were detected only in trace amounts of ≥0.1 mg/g dw.

In *A. montana* cv. Arbo flowers, all fourteen SLs were identified. At the flowering stage, the predominant H derivatives were 2-methylbutyrylhelenalin (HMB) and isovalerylhelenalin (HIV) (*m/z* 347.19), and isobutyrylhelenalin (HIB) (*m/z* 333.17) in the amount of 9.42 mg/g dw and 4.06 mg/g dw, respectively. In the buds, the content of the lactone was on average two times lower in both *A. montana* taxa (*A. montana* and *A*. *montana* cv. Arbo) than in the full flowering stage.

### 2.3. Influence of Flower Development Stages of SLs

**Figure 3:**
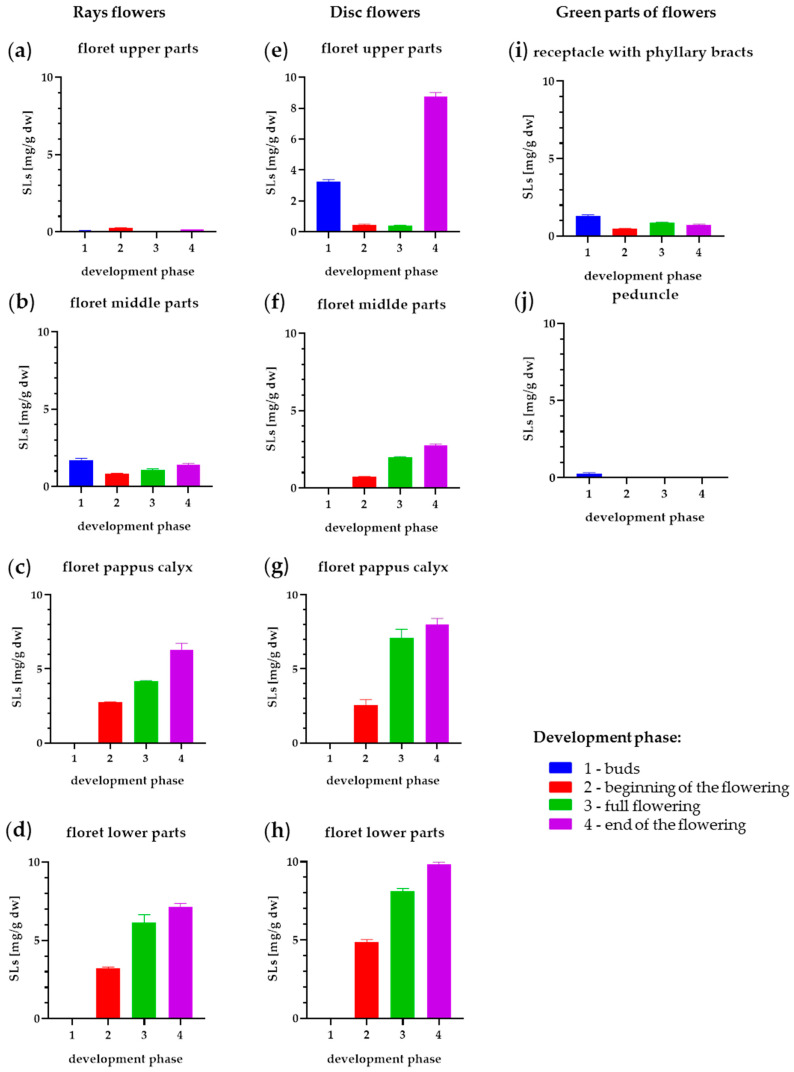
Concentration of helenanolides and dihydrohelenanolides ± SD (mg/g dw) in different parts of ray flowers (**a**–**d**), disc flowers (**f**–**i**), and green parts of flowers (**e**–**j**).

Analyses conducted on the whole flowers of the studied plants allowed us to select the most efficient taxon that produces the highest amount of SLs. Therefore, in further research, the most productive German variety of arnica, *Arnica montana* cv. Arbo, was used. Analysis of the flower parts in subsequent periods of development revealed significant differences in SL content (Tables S3–S6 and Figure 3). It has been observed that, generally, the concentration of SLs increases with the maturation of flowers in all flower parts; however, there were a few exceptions. In the analyzed extracts from the combined parts of the flower receptacle with phyllary bracts and the peduncle, the SL content remained at a similar level for each of the examined stages of flower development and ranged from 0.47 to 1.30 mg/g dw, with the highest content recorded in the buds. In extracts from the florets’ upper parts, SLs were present from the moment of formation of the flower bud, but only in ray flowers, which ripen faster. In the next stages of development in the floret upper parts, the amount of SLs decreased, and at the end of flowering, it increased again to 8.78 mg/g dw. In disc flowers located in the buds, SLs were not found in the florets’ upper part. The peduncle practically contained no SLs, regardless of the development phase. The largest amount of SLs occurred in the parts related to seed formation, namely in the florets’ middle parts, in which values of 4.89–8.10 mg/g dw were recorded in disc flowers and 3.22–7.15 mg/g dw in ray flowers, depending on the phenological phase of development. Interestingly, SLs in the ovary were not present at the beginning of flowering. Surprisingly, the floret pappus calyx turned out to be another productive part of the flower, in which the SL content increased with development from 2.75 to 6.31 mg/g dw in disc flowers and from 2.58 to 7.99 mg/g dw in ray flowers. The results indicate a linear increase in SL concentrations in the florets’ lower parts and the florets’ pappus calyx with the maturation of the flower. Two-times lower SL synthesis was observed in the florets’ middle parts, containing 0.72–2.78 mg/g dw in disc flowers. In the same part of ray flowers, the highest SL content, 1.73 mg/g dw, was recorded in the buds phase; then, their concentration decreased twofold and then increases to the initial level of 1.44 mg/g dw in the last flowering phase.

Considering the inflorescences as a whole part, we observed a high structural variety of SLs derivatives, in which all of the fourteen analyzed metabolites were identified. Specific parts of the flower show some differences in the ability to synthesize and accumulate individual compounds. The largest amount of HMB and HIV derivatives were noted in the florets’ upper parts. The composition of derivatives in ray flowers was similar, although, in addition, HIB was detected in higher content.

The richest spectrum of SLs was observed in the florets’ lower parts and in the florets’ pappus calyx. The dominant derivatives found in the florets’ pappus calyx, middle, and lower parts, were HMB/HIV and HIB. Additionally, in the florets’ middle and lower parts, significant amounts of methacryloylhelenalin (HM) and tigloylhelenalin (HT) derivatives were observed.

Considering the whole inflorescences of *Arnica montana* cv. Arbo (Tables S7 and S8) and the differences in their ability to the synthesis of SLs in two types of flowers, the total yield of lactones can be estimated at 60.87% for disc flowers, 32.9% for ray flowers, and 6.22% for the green parts of the flower, i.e., the receptacle with phyllary bracts and peduncle. In the green parts, the highest synthesis of SLs was observed in phyllary bracts and was equal to 5.78%; only 0.44% was observed in the receptacle, while in the peduncle, SLs were not found.

### 2.4. Structure and Morphology of Flower Trichomes

**Figure 4:**
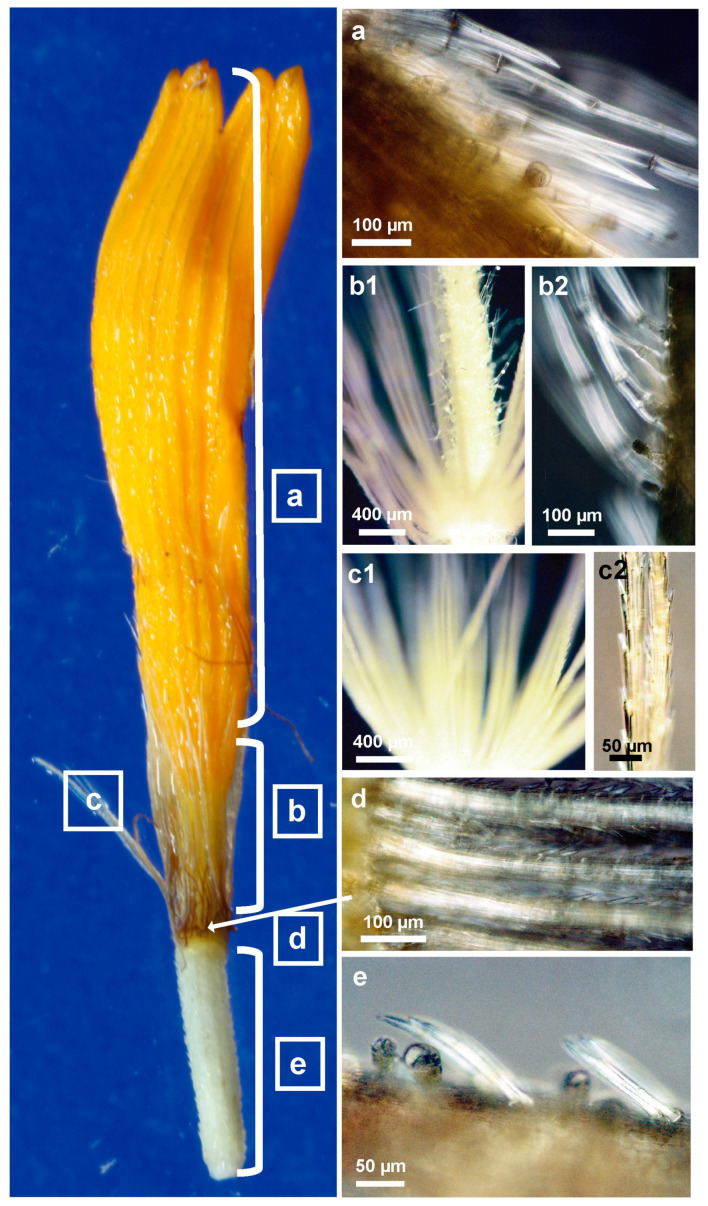
The image of single disc floret from *A. montana* cv. Arbo flower heads. (**a**–**e**) Polarization of glandular and non-glandular trichomes results from fatty aldehydes, inulin, secondary metabolites, or cellulose. (**a**) the upper part of floret; (**b1**,**b2**) the middle part of floret; (**c1**,**c2**) pappus calyx with single bristle; (**d**) the middle part of the floret with bristle whorl; and (**e**) the lower part of the floret–ovary with twin covering and glandular trichomes.

*Arnica montana* is a plant species characterized by a compressed racemose inflorescence called capitula, which comprises of individual flowers that are divided into disc and ray florets. The individual floret is placed on a common receptacle with one per florets’ non-glandular trichome, which are colored purple. The florets’ corollas are characterized by the presence of non-glandular and glandular trichomes, mainly on the abaxial side, the distribution and density of which are specific to the part of the floret. The glandular trichomes found on the floret are exclusively of the capitate double-celled glands type of Asteraceae (Figure 4). Prior to secretion, these trichomes are sessile and subsequently become elongated, exhibiting 4–5 cell layers with a short stalk. The ovary is distinguished by the densely packed capitate double glandular trichomes and twin covering hairs, which are exclusively present on this part of the floret. Above the calyx whorl, specifically on the fused narrow section of the petal tube or tabularized corolla, which includes the perianth, designated as the middle part of the floret in this study, there exist both glandular and non-glandular trichomes. The non-glandular trichomes in this area are very lengthy, bent upward, and composed of 4–5 cells. On the upper part of the floret, specifically on the expanded section of the corolla or throat, located above the narrowed part of the fused tube of petals, the occurrence of secretory glands and covering hairs of the same type is less frequent; however, on the pointed triangular ends of the petals or lobes, there are 3–5 large secretory glands situated on the central vascular bundle. Furthermore, conical papillae are present on the adaxial surface of the florets’ upper petals.

The generative organs of the floret, specifically the stamens comprising the filament and anther sacs held together by the connective tissue, which consists of parenchyma cells, do not possess any secretory glands. Similarly, the style and stigma also rarely exhibit small glands observed only on the abaxial surface of the pistil. Two types of secretory glands are observed on the phyllary bracts and stalk, namely capitate trichomes on short and long stalks, whereas abundant 2–4-celled uniseriate covering hairs are present on the edges of the involucre bracts and on the axial and abaxial sides. The same types of glandular and non-glandular trichomes are also found on the peduncles. Asynchronous cell divisions in the biseriate long-stalk capitate trichomes with round heads are a characteristic feature of this type of gland.

### 2.5. Histochemical Analysis

In this study, we investigated the properties of fatty acids that form esters with H and DH (short-chain fatty acids), the first four of which are water soluble and have a unique scent. H is a member of a class of compounds with two alkylating centers, one of which is an exocyclic methylene lactone and the other is a conjugated cyclopentenone with an unsaturated ketone group. DH may be less active in some reactions because it lacks one reactive center, owing to its saturated methyl lactone rather than an exocyclic methylene lactone group. Furthermore, the presence of longer carboxylic acid ester chains can complicate in situ identification by acting as allosteric hindrances to the detection of the reactive group in the SLs structure; however, both H and DH can be detected by histochemical methods because they contain an endocyclic unsaturated ketone group.

**Figure 5:**
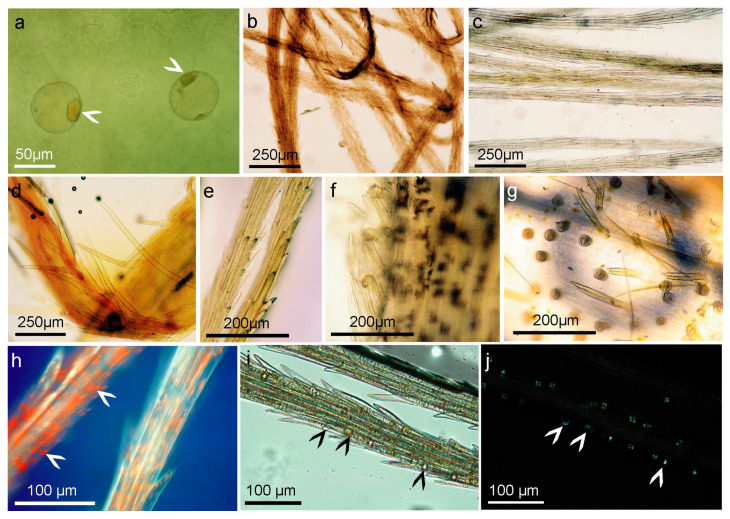
The histochemical analysis of *A. montana* cv. Arbo single florets from the flower head. (**a**) Zimmermann’s reaction for methyl ketones and aldehydes—positive reaction visible in the head of glandular trichomes (arrowheads); (**b**,**c**) Zimmermann’s positive reaction is visible in the bristles of the pappus. (**b**) Ray floret, (**c**) disc floret; (**d**–**f**) the Legal test for methylene and methyl ketones, (**d**) phyllary bracts,(**e**) pappus bristle, (**f**) middle part of the floret; (**g**) test for ketones/aldehydes with 2,4-dinitrophenylhydrazine on the lower part of the ovary; (**h**) the bristle of pappus stained with Schiff reagent in UV light excitation at 395 nm, using a Zeiss barrier filter (FT 396, LP 420 barrier filter) showing the presence of aldehydes in the lumen of inulin vacuoles (arrowheads); (**i**,**j**) inulin spherocrystals in dried bristles of pappus (arrowheads), in white transmitted (**i**) and polarized light (**j**).

A variety of reactions, including the Zimmermann reaction for methyl ketones (Figure 5a–c), the Legal test for methyl and methylene ketones (Figure 5d–f), and the 2,4-dinitrophenylhydrazine assay were applied (Figure 5g). Potassium metabisulfite and Schiff’s reagent were used to localize aldehydes in the plant tissues [41].

**Figure 6:**
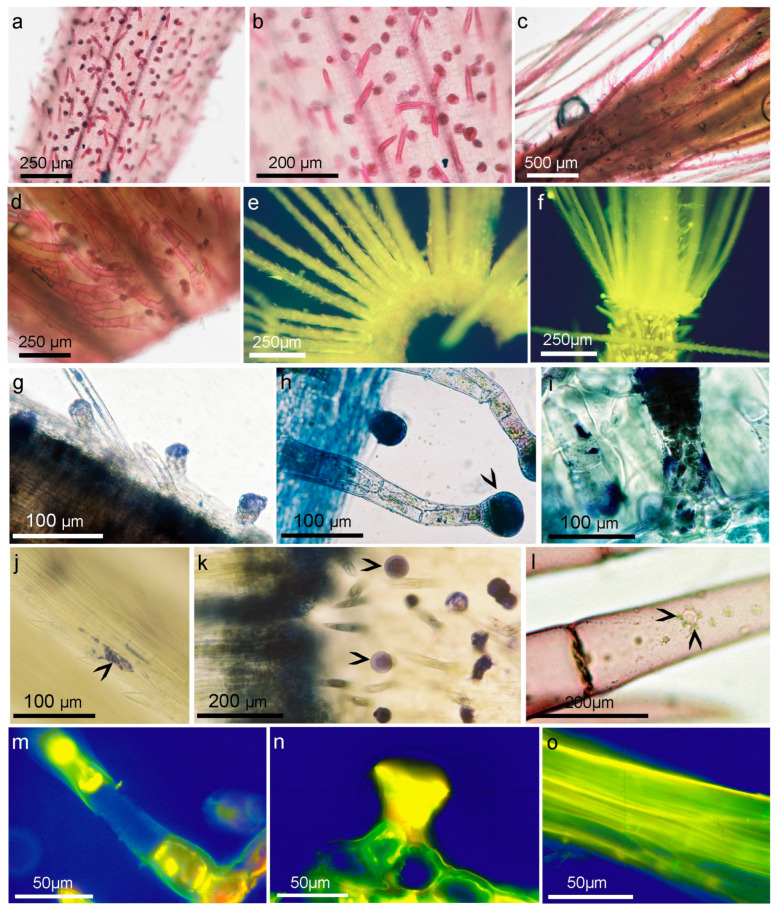
The histochemical analysis of *A. montana* cv. Arbo single florets from the flower head, peduncle, and leaf. (**a**–**d**) Schiff’s reagent for aldehydes, (**a**,**b**) ovary with stained glandular and twin covering trichomes. (**c**) Bristle of pappus, (**d**) glandular, and non-glandular trichomes on the middle part of the floret; (**e**) fluorescence in blue light excitation at 450–490 nm, using a Zeiss barrier filter LP (520) of pappus whorl of bristles treated with AlCl_3_ for lactones; (**f**) green autofluorescence of pappus, glandular and non-glandular trichomes of dry achene in blue light excitation at 450–590 nm, using a Zeiss barrier filter LP (520); (**g**–**k**) reaction with NADI for cytochrome oxidase, (**g**) glandular and twin-celled trichomes on the ovary, (**h**) glandular trichomes on the peduncle, (**i**) glandular and non-glandular trichomes on phyllary bracts, (**j**) pappus bristle, (**k**) bristle whorl of pappus, non-glandular and glandular trichomes (arrowheads) on the ovary; (**l**) succinate dehydrogenase activity in covering trichomes of leaf, and oil body surrounded with leucoplast (arrowheads); (**m**–**o**) immunolocalization of farnesyl antibody (yellow-green fluorescence), (**m**) covering trichome on petal epidermis, (**n**) glandular trichome on adaxial leaf epidermis, (**g**) pappus bristle.

The tests revealed two sites of methyl and methylene ketone localization, biseriate secretory glands and calyx pappus, as well as two types of non-glandular trichomes that stained less strongly. The most selective action in relation to epidermal formation was that of Schiff’s reagent, which indicated the presence of fatty aldehydes (terpenes), decomposed fatty acids, oxidized lipids, and perhaps esterified saccharides, such as oligofructans, and some phenolics (Figure 6a–d).

These findings revealed the details of the cell walls of these epidermal structures. The walls of the non-glandular and glandular trichomes were thick and formed in a similar manner. They have a cellulose skeleton structure that emits delicate blue UV fluorescence and are likely enriched with unsaturated lipids and secondary compounds, as well as specific sugar polymers, such as pectin esters, algins, and fructans. *A. montana* tissues accumulate inulin, which is composed of fructose chains that vary in length and end with a glucose residue, and an oligofructosidic mixture of shorter fructose chains that end with glucose or fructose.

Small vesicles are secreted on the surface of the cuticle of the covering and glandular hairs, the nature of which is probably resinous or polyterpenoid but may also be salt accumulation or mucilage, which makes the organ sticky.

The fluorescence of flower fragment trichomes under UV light was slightly blue, which indicates cellulose fibers, and green or light green under blue light (Figure 6f). However, the use of alkannins, which dye lipids (fats, cutin, suberin, and wax), essential oils, resins, and rubber, suggests that their walls also contain lipid derivatives. The secretory and covering glands were stained red under the influence of alkannin, but the lower calyx contained lipids in the bristle whorls and oil bodies scattered along the longitudinal walls of the pappus. Interestingly, these trichomes and bristles turned brown under the influence of Oil Red O, indicating that the glycerides present in them did not include saturated fatty acids; rather, their oxidation products, unsaturated fatty acids, or intermediate secondary metabolites were synthesized.

All types of hairs present or existing on flowers show polarization, and in the pointed ends of the non-glandular trichomes and bristles of the pappus, inulin spherocrystals in vacuoles can be detected by this method, which in living cells is visible after staining with Schiff’s reagent but only under UV light (Figure 5h–j). The cells and walls of both types of trichomes were stained with Schiff’s reagent, which may suggest that various natural aldehydes are also placed along plasmalemma or exported to the apoplast and cell wall. It is possible that the presence of fructans of short length may be responsible for the registered polarization, or that it is the result of a regular arrangement of cellulose microfibrils encrusted with fructans, lipids, or even calcium. The most abundant sesquiterpene lactone ester in *A. montana* flowers is the 2-methylbutyric acid ester, which, like most of the other derivatives and together with H, is a chiral compound that rotates in polarized light. Unfortunately, our results showed that these features did not correspond to the one light-polarizing substances found in the trichomes.

In flower tissues, the distribution of terpenes and lipids was studied using the NADI reagent for cytochrome oxidase, an enzyme involved in the final metabolism of mono- and sesquiterpenes (Figure 6g–k). The heads of glandular trichomes were intensely stained. In non-glandular trichomes, much smaller terpene deposits occurred along the cell walls, where 3–6 droplets of terpenes were locally found in single cells. In the twinned covering trichomes characteristics of the ovaries, blue staining was observed along their tangential walls, where plasmodesmata were present, and at their pointed ends. In the pappus, the strongest reaction was observed in the bristle-fused whorls and basal parts of individual bristles. Less frequently, NADI-stained terpene oil drops were scattered along the longitudinal sidewalls of the bristles and at the ends of the pointed setae. The neotetrazolium reaction was used to demonstrate the presence of succinate dehydrogenase, which stained light-blue lipid vesicles surrounded by an assembly of several structures resembling a leucoplast with other smaller organelles (Figure 6l).

Succinate dehydrogenase dehydrogenates succinate by removing two hydrogen atoms and transforming it into fumarate, which acts as an electron acceptor by participating in oxidation reactions. This dehydrogenase has a flavoprotein prosthetic group similar to acetyl-CoA dehydrogenase. Both dehydrogenases are strongly bound to mitochondrial enzymes. Its association with leucoplasts and other smaller organelles occurring in cells around lipid vesicles suggests its importance in lipid catabolism and likely in the biosynthesis of sucrose and secondary metabolites.

Reagents used to detect SLs using thin-layer chromatography, as reviewed by [42,43], have also been tested and applied to plant tissues. Of the reactions used, only a few—p-dimethylaminobenzaldehyde, cinnamic aldehyde, vanillin, the 2,4-dinitrophenylhydrazine assay (Figure 5g) EDTA and AlCl_3_ (Figure 6e)—gave visually good reactions in the case of glandular and non-glandular trichomes and the pappus, which may indicate the accumulation of H and DH derivatives both in the heads of the secretory glands, inulin vacuoles as well as in the apoplast and cell walls of the pappus and non-glandular trichomes.

Protein prenylation involves the transfer of either a farnesyl or geranyl moiety to the C-terminal cysteine of a target protein. The anti-farnesyl transferase antibodies are commercially available, which enabled us to study the protein prenylation pattern in flowers of *A. montana* cv. Arbo. Our study showed the presence of anti-farnesyl antibodies in the heads of glandular hairs and less intense antibody signals along the longitudinal walls of the pappus bristles (Figure 6m–o). This pattern of antibody localization in the papilla resembled the accumulation sites of lipid droplets recorded with NADI and alkannin. Glandular trichomes in the active secretory state are the source of very intense signals, whereas in non-glandular trichomes, this signal is weaker.

The involvement of non-glandular trichomes in lactone biosynthesis was indicated by a comparison of the number of trichomes present on the seed coat of *A. montana* and *A. montana* cv. Arbo achenes (Table 2). The cultivar synthesizes more than 58% of SLs in its flowers compared to the wild species cultivated in a botanic garden but originated in the wild. Comparing the number of twinned covering trichomes present on the ovary of both taxa, it was found that their number in the cultivar was 35.45% higher than that in the wild species, while the number of glandular hairs was 16.7% higher; however, the diameter of glandular trichomes was 6.65% larger. These results indicated that non-glandular trichomes may also be involved in the biosynthesis of SLs.

## 3. Discussion

### 3.1. SL Accumulation in Flower Heads in Arnica Taxa

Herein, we have studied esters of H and DH in three *Arnica* taxa: *Arnica montana* L. and its variety *Arnica montana* cv. Arbo and *Arnica chamissonis* Less. The tested plant material, consisting of flowers and their parts, came from controlled field cultivation. The maximum yield of flowers was obtained in the 2nd and then in the 3rd year of cultivation, and from the 4th year, it was successively reduced. After 3 years of growth, the flowers were subjected to qualitative, quantitative, and histochemical analyses in various phases of development. The most productive in SLs was *A. montana* cv. Arbo. A two-times lower amount of SLs were observed in typical species. *Arnica chamissonis* contain small amounts of H and its derivatives, while esters of DH were practically absent.

In ray flowers, the total SLs content was two times higher than in disc flowers, the dry weight distribution was 33% for disc flower and 61% for ray flower. The reason is likely the fact that ray flowers ripen earlier than disc ones. A similar trend was observed by Douglas et al., dry weight distribution was 23% for disc flower and 57% for ray flower [27]. The SLs content in ray flowers, two times higher than in disc flowers, may result from their earlier maturation. The flowering sequence within individual flowers runs centripetally, i.e., from the edge of the basket towards the inner whorls. Ray flowers mature faster than disc flowers, have a larger modified corolla, and are physically bigger.

Within the studied taxa, we observed the differences in the helenanolides and dihydrohelenanolides profiles. In flowers of *Arnica montana*, DH dominated over H in flower buds, while at the end of flowering, this ratio equalized. In *A. montana* cv. Arbo for each of the examined stages of flower development, H esters predominated over DH with a ratio of 1.4, and during the flowering, it increased two times (ratio equal to 2.9). Populations of *A. montana* in Central Europe can be distinguished from the European chemotype, in which H esters predominate, called *A. montana* subsp. *montana*, and the Spanish chemotype, in which DH esters predominate, known as *A. montana* subsp. *atlantica* [28,44].

Spanish populations of *Arnica* growing in mountainous areas produce the highest amounts of SLs in meadows, less on heathlands, and the least on peatlands. The highest ratio H/DH was recorded for meadow populations. In the *A. montana* populations of Central Europe (total 9.9 mg/g SLs) and Spain (total 8.08 mg/g SLs), the total SL levels were similar, while the content of the particular derivative differed. In the samples from Central Europe, the H esters concentrations (total H 8.76 mg/g dw) were significantly higher than those of DH esters (total DH 1.14 mg/g; H/DH ratio 7.68). A commercial sample from Spain contained H esters at a lower level (total H 1.00 mg/g) than DH esters (total DH 7.08 mg/g; H/DH ratio 0.14) [28].

We have noticed that the concentration of SLs rises during plant growth, in accordance with the literature [27,30]. Therefore, the optimal time to collect pharmacopeial raw material is after flowers have withered. Sugier reported that regardless of the method of establishing plantations, the raw material obtained at the end of flowering heads accumulated the highest content of lactones (average 1.17%), while the lowest (average 0.92%) was found in the raw material that was collected in the yellow bud phase [30]. These results are also consistent with the reports of Douglas et al., according to which the content of SLs in arnica flower heads systematically increases from the phase of flower buds to the moment of reaching seed maturity [27].

In the studied taxa, differences in the occurrence of helenanolides and dihydrohelenanolides were observed. The proportions between H and DH derivatives for our native population of *A. montana* from the Karkonosze Mountains (Hala Izerska sites) do not resemble either the Central European group or the Spanish type. The characteristic for the Arbo cultivar was a predominant content of H derivatives, though it was not as high as in the high-altitude populations of *A. montana* subsp. *montana*, as analyzed by Perry et al. The authors reported that the increased intensity of UV radiation in high mountain conditions affects the synthesis, but only of H derivatives [28]. Genetic studies by Vera et al. based on the amplification of two polymorphic chloroplast markers of intron *rps16* and the spacer *ycf4-cem* for populations allowed for the detection of two haplotypes that were different from those previously described in Europe, showing significant biochemical differences and occupying different environments [45]. For example, the content of 6-O-isovaleryl helenalin in *A. montana* cv. Arbo exhibits a highly significant correlation with climatic factors [31]. Based on the proportion between H and DH esters, we can conclude that the Polish population is not related to the populations occurring in Spain, but rather represents the Central European type; however, it should be emphasized that the plant growing conditions, such as temperature, can significantly modify SL profile. An example may be the cultivation of *A. montana* documented by Bauer in New Zealand, where plantations located at low altitudes did not bloom, while their transfer to higher mountain areas resulted in flowering, due to vernalization required for this species [46].

Seemann et al. have denoted the ratio to H/DH in ten wild populations of *A. montana* collected in meadows and heathlands at different altitudes and climatic conditions in Germany [31]. The ratio of helenanolides to dihydrohelenanolides was in the range 1.5–2/1, regardless of the different environmental conditions of the sites. In our studies on Arbo cultivar, this proportion at the flowering stage was 2.9–3.7/1. According to the European Pharmacopoeia, inflorescences of the “Arbo” type corresponds to the Central European chemotype [47].

Schmidt et al. observed that the H esters content of young *A. montana* plants decreased to almost zero within about 6 weeks of the beginning of leaf formation, while the DH esters content was gradually increasing, indicating the presence of a hydrogenase system that converts helenanolides to dihydrohelenanolides [48]. In the leaves of our taxa, H esters were not practically observed.

SLs are the most important secondary metabolites responsible for the anti-inflammatory properties of the *Arnica* plant [49]. The anti-inflammatory activity of H esters is much greater than that of DH esters, but DH esters are less sensitizing and are, therefore, more valued in the pharmaceutical industry. The diversity of chemotypes allows for the selection of plant material with a composition suitable for certain applications or pharmaceutical needs [45,50]. Apart from strong anti-inflammatory activity, H is known to treat minor injuries. Water and ethanolic extracts from *A. montana* flowers also possess anticancer activity [51]. H also has antitelomerase activity in a variety of cancers and tumor tissues; it shows a cytotoxic effect in the T47D breast cancer line and decreases the expressional values of the hTERT gene in a dose-dependent manner [52]. H promotes apoptosis by the release of cytochrome c from mitochondria and arrests the G2/M-phase of the cell cycle [53]. Potential applications of H and DH are reserved in over 100 patents related to medical and cosmetic uses, from shampoos and wound healing accelerators, substances eliminating the effects of bites, to specifics for capillary skin. Due to the stronger allergenic effect of H, there may be indications for the use of DH, which can be obtained by picking flowers at an earlier stage of development. Studies by Widen et al. suggest that α-methylene-γ-butyrolactone contributes most significantly to the NF-κB inhibition in the case of simplified H analogues [54].

*Arnica montana* L. is a protected species, and its natural habitats are constantly disappearing. The places of occurrence of arnica in Poland are located in the northwest and southeast site of the country. The main causes of the disappearance of the species reported by Luijten et al. and the [55] are exploitation by the pharmaceutical industry, habitat loss partly due to agriculture, land abandonment, and reforestation [56]. In Poland, the succession of plant communities and flowers eaten by deer in mountain areas is also important. To preserve the natural resources of this species, it will be necessary to apply appropriate conservation actions [57]. In our experiments, plant propagation was carried out via in vitro culture. Optimal growing conditions allowed us to maintain plants with good health status and that were free of diseases and pests. Such plants could be used for conservation activities of the species by enriching the natural site with seeding or semi-crops. Subseeding is successfully implemented in Germany [57]. Our studies indicated the proper climatic and soil properties for the cultivation of arnica and that these conditions can be met in western Poland. Undoubtedly, these crops would also help maintain natural populations if used as sources of germplasm and for ex situ conservation, but they would also be of interest to the pharmaceutical industry [58].

There are many hypotheses about the location of biosynthesis of SLs. Some suggest that they are produced in glandular trichomes that cover the surface of leaves, stems, flowers, and ovary [46]. The explanation is that SLs could not be isolated from *A. sachalinensis* (subgenus Andropururea), which is characterized by the absence of glandular trichomes [33]; however, the abovementioned presumption had not been yet confirmed by proper experiments. Therefore, we decided to separate the flowers into appropriate parts to determine the place of SL biosynthesis in flower heads, additionally studying the metabolites profile changes depending on the phenological phase. The synthesis of SLs takes place in the flower parts, which are associated with the formation of seeds: the florets’ lower and middle parts, and the pappus calyx. This is the first report on the content of secondary metabolites in the florets’ pappus calyx. It turned out that the SL content in this part of the flowers is significant and ranges from 2.58 to 7.99 mg/g dw, depending on the growing stage.

The florets’ lower parts contained 60% of lactones in the case of ray flowers and 46% in the disc flowers. Less amount, equal to 19% and 25% of the total lactones were contained in the florets’ middle parts ray and disc flowers, respectively, while the florets’ pappus calyx contained 10% and 23% of all SLs, and the petals contained 11% and 6% of the total amount of SLs in both types of flowers, respectively. The analyzed stamen and stigma did not reveal the presence of SLs. Interestingly, only three derivatives are synthesized in a significant amount in the florets’ middle parts: HMB, HIV, and HIB. Due to the high content of individual derivatives, the obtained results can be used to select a plant material rich in specific esters of H or DH. The composition of the esters varied depending on the extracted material. This is valuable information for the pharmaceutical industry should the manufacturer wish to obtain a certain metabolite with specific properties. HMB and HIV increased with flower maturation in each of the flower parts studied, except for the upper part of the flowers, where the relationship was not linear.

Interestingly, the content of metabolites in the florets’ pappus calyx increases as the flower matures, which suggests that its biosynthesis is related to the specific functions of the plant. Within the whole flower, the majority of SLs were synthesized in the florets’ lower parts. The largest accumulation in flower parts related to seed formation may indicate the defensive function of SLs. Several types of hairs were identified in *Arnica*: glandular, protective, longitudinal trichomes. Mature arnica achenes are dispersed by wind and gravity, and the trichomes together with pappus calyx significantly increase their spreading distance, adhesion to the soil, and the mass of water taken up and moisture. Perhaps the SLs found in the floret pappus calyx and floret lower parts are intended to defend against microorganisms. It is possible that they are released into the substrate to increase the chances of survival. However, this should be confirmed in another research study. In flowers of *Arabidopsis*, monoterpene, and STPS were not present in the flower petals; instead, their expression is limited to the stigma, anthers, nectary, and sepals, suggesting that the volatile terpenoids synthesized in *Arabidopsis* flowers may act not only as an attractant of short-range pollinators, but they may also be important for the defense of flower tissues by inhibiting the growth of pathogenic microorganisms or repelling herbivores from particularly sensitive flower parts [14].

To produce these valuable metabolites on an industrial scale, it is necessary to fully understand the biosynthesis pathway and its regulation in tissues, which should be verified.

### 3.2. Histochemistry Studies

In plants, fructans serve as reserve carbohydrates and play several important roles in enhancing plant tolerance to cold and drought [59]. Using polarized light, inulin crystals similar to those observed in *Arnica* were detected in the root cortical parenchyma of *Pombalia calceolaria* (Asteraceae), a plant used in folk medicine in Northeast Brazil [60]. Silva et al. recognized fructans as a source of energy and protection against drought and extreme temperatures in *Dimerostemma vestitum* (Asteraceae), where inulin-type fructans were found in all organs except the leaves and secretory structures [40]; however, in agave, fructans were present in all organs [61,62]. Fructans may act as signaling molecules, such as microbe-associated molecular patterns (MAMPs) and damage-associated molecular patterns (DAMPs), and may also be involved in osmoregulation during flowering [62,63,64,65]. Recently, the antioxidant activity of inulin was demonstrated both in vitro and in vivo. In vitro, the radical-scavenging antioxidant effect of DPPH and ABTS, and the iron-reducing capacity of inulin were lower than those of vitamin C [66]. This activity in *A. montana* protects unsaturated lipids and intermediates from oxidation.

The ability of fructans to rapidly polymerize and depolymerize [67], and their adherence to cell membranes inside and outside cells, as in *Campuloclinium chlorolepis* [68], provides evidence that fructans may play important roles in membrane stabilization and adaptation to stressful environmental conditions. The detection of fructans outside the plasmalemma in *Arnica* plants may indicate the incorporation of these compounds into the cell wall, which increases water absorption capacity and protects the seedling from drying out. In addition, fructans may increase calcium and magnesium accumulation [69], thereby facilitating the uptake of these essential elements by developing plants.

The distinctness of the glandular and non-glandular trichome cell walls we demonstrated in *A. montana* may be related to the specific role of sugar polymers in the accumulation of terpenes, which was confirmed by [37] on the remodeling of cell wall subdomains of *Cannabis* during trichome development. Cavity biogenesis is linked to the formation of a subcuticular wall rich in homogalacturonan and -1,5 arabinan zones. Droplets of metabolites are packaged in growing cavities by glycoproteins, which provide an emulsifying physicochemical interface between the lipophilic metabolites and residual hydrophilic apoplasts. In *A. montana* glandular trichomes, droplets present in short stalk cells stain with vanillin at the beginning of the secretory stage, which indicates the presence of fructans. However, vanillin staining is not specific and shows helenalin and steroids.

The ability to synthesize lipids and polyphenols in *Inula helenium* covering hears tissues was noted by [70], who believed that in addition to their protective role, trichomes may have a secretory function, which was evident after histochemical assays [71]. Since then, there have been a growing number of reports on new functions beyond the physical protection of non-glandular trichomes connected to the chemical interactions of plants with the environment, supplementary to glandular trichomes [72,73]; however, [74] stated that the volatile terpene compound content in *Chrysanemum* leaves was significantly correlated with the density of glandular trichomes but not with the T-shape covering trichomes. This finding supports the hypothesis that glandular trichomes are responsible for the emission of volatile terpenes. It is worth noting here that the SLs of *A. montana* do not belong to the volatile components, and the esters present in the tissues, especially the longer fatty acids, should be dense non-volatile oils; as such, they could thicken the fraction of essential oils in glandular trichomes, or because of their toxicity, they could be deposited in the cell walls of non-glandular trichomes or inulin vacuoles.

Pershev et al. [75] proposed model for the role of fructans molecules contracting oxidative stress by including vacuoles as a site for antioxidant processes. The availability of membranes for strong interactions with fructans [76] and synergistic interaction with phenols, as deducted [75] allowed cooperation between pools of both compounds in vacuoles. The authors distinguish within the vacuole two areas the inner space near the tonoplast with a higher concentration of fructans and the central lumen of the vacuole, which is dominated by phenolic compounds. The images we obtained of numerous vacuoles that reveal the presence of aldehyde/ketone-like compounds under UV light confirm the described model.

The visible association of oil droplets with leucoplasts and probably much smaller glyoxysomes and mitochondria after staining for succinic dehydrogenase with Neo-T resembles that introduced by [77]. Triacylglycerol hydrolysis is performed by lipase, the free fatty acids are imported to glyoxysomes, and subsequently formed succinate can be converted to sucrose in mitochondria. Oil bodies are responsible for antifungal oxylipin formation but may also provide active secondary compounds, as proposed by [77] or deliver substrate for fructan synthesis.

Studies conducted to date on the importance of post-translational modifications of lipids have highlighted their role in signaling plant resistance to disease and in response to various stresses. The involvement in stress responses and developmental processes has been demonstrated through the analysis of abscisic acid (ABA) susceptibility and response mutants. Along with increased drought tolerance, this mutant was sensitive to high temperatures [61,62]. It has also been proposed that another prenylated protein and cytochrome P450 that catalyzes the conversion of casasterone to brassinolide is responsible for pleiotropic effects in the *era1* mutant [78]; however, in the case of *A. montana*, prenylation of proteins could influence formation of SLs. 

Although relatively simple, these modifications are multifunctional and have tremendous potential to regulate protein function in a variety of ways, including localization to membranes or specific microdomains within them, affecting protein activity, complex formation, and other interactions [79].

The intense signal on anti-farnesyl antibodies in the glandular and non-glandular trichomes of *A. montana* may also be due to the high metabolic activity of the trichome cells and intensive membrane transport between organelles by vesicle-channel carriers, possibly related to the export of SLs or fructans outside the cell in the form of vesicles. Prenylation is crucial for the function of a variety of membrane-bound enzymes, such as the Ras, Rho, and Rab families of small GTPases responsible for cargo delivery [80,81,82]. Soluble N-ethylmaleimide-sensitive-factor attachment protein receptor (SNARE) mediates vesicle fusion with the target membrane and is a farnesyltransferase substrate that regulates docking and SNARE-mediated exocytosis [83,84]. This mechanism may also be responsible for the transfer of vesicles into the cell and can mediate exocytosis and endocytosis with intermediates that are synthesized in the lower stalk cells of glandular trichomes.

In summary, the histochemical analyses showed the specificity of the chemical composition of the pappus calyx and epidermal extensions, which were characterized by the presence of inulin, lipids, enzymatic activity of succinate dehydrogenase and cytochrome oxidase, and the presence of SLs, as proven by UHPLC-MS tests.

## 4. Materials and Methods

### 4.1. Plant Material

The tested plant material, consisting of flowers and their parts, came from controlled field cultivation. The whole florescence of three *Arnica* taxa in two stages of growth, namely buds and flowering flowers, were examined. The biosynthesis of SLs in various flower parts, divided into ray and disc flowers, was checked, namely, florets’ upper, middle, and lower parts, receptacle with phyllary bracts, and peduncle of *A. montana* cv. Arbo, which are in four stages of development (buds, beginning of the flowering, full flowering, and end of the flowering). Extending the analysis, the florets’ upper parts of *A. montana* cv. Arbo was divided additionally by petal, pistil, and stigma. The plants were grown in the botanical garden of the University of Wrocław, Wrocław, Poland, 51°6′57.444″ N, 17°2′52.274″ E. Seeds of *A. montana* L. and *A. chamissonis* Less. came from the Hala Izerska site at an altitude of 840–880 m n p m. (Izerskie Mountains), Sudety, Poland while the seeds of the *A. montana* cv. Arbo were obtained from a private planter from Germany. Plant propagation was carried out in vitro culture. Then, the plants were acclimatized under greenhouse conditions and grown on experimental plots, using the substrate described by our previous research [85].

### 4.2. Reagents

The applied standards of (−)-α-santonin and helenalin were obtained from Merck Millipore (Warsaw, Poland) and Cayman (Ann Arbor, MI, USA), respectively. Ethanol, methanol, formic acid, and water were purchased from Merck Millipore (Warsaw, Poland), and enkephalin-leucine was obtained from Waters (Milford, MA, USA).

### 4.3. LC-QTOF-MS Quantitative Analysis and Method Validation

Extracts and calibration standards were prepared using previously validated and described methods [27,28,34].

For a quantitative analysis of SL linearity, accuracy, precision, and recovery, as well as limits of detection (LOD) and quantification (LOQ) were determined.

#### Method Validation

- Linearity

Calibration curves were prepared by dissolving stock solutions in methanol with 0.1% of formic acid to yield the concentrations of: 0.2; 0.4; 0.8; 1.0; 2.0; 4.0; 8.0; and 10.0 µg/mL. Calibration curve linearity was determined by calculating the coefficient of correlation (acceptable threshold: ≥0.995) and by conducting *t*-test for slope significance. The working range of the calibration curve was determined by assessing the homogeneity of variances of the lowest and highest calibration levels with the Snedecor *F* test. The calculated *F* value should be equal to or below the expected *F* value. The following formula was applied to the established *F* value: *F*_cal_ = the highest relative standard deviation (RSD) for the calibration level/the lowest RSD for the calibration level.

- Accuracy and precision

Spiked fresh leaves at concentrations 1.0 µg/mL were used to determine accuracy and precision. The coefficient of variation (CV) for each level should be below 15%, which is consistent with EMA and FDA guidelines for validating the analytical method.

- Recovery

Recovery rates were evaluated at a concentration of 1.0 µg/mL and prepared by adding 50 µL of proper working standard solution to 1 g of helenalin leaves samples. The following equation was applied: (response obtained for spiked leaves − response of non-spiked leaves)/response of working standard solution diluted in ethanol × 100. Recovery rates should be in the range of 80–120%.

- Limit of detection and limit of quantification

The limit of detection (LOD) and the limit of quantification (LOQ) were determined based on the standard deviation (SD) of the response and the slope of calibration curves. The SD values of the response were estimated as the residual standard deviation of a regression line. The following formulas were applied: 3.3 × SD/slope for LOD and 10 × SD/slope for LOQ.

The obtained t_cal_ value of slope significance nt *t*-test was 80.80 and was above the critical t value (t_crit_ = 2.02). The calculated *F* value was 1.30. It was obtained for *n* = 6 and *α* = 0.05 and was below the expected *F* value (*F*_crit_ = 5.05), which is indicative of the correctness of the calibration curve range.

### 4.4. Extraction of SLs

The extraction procedure was carried out by grinding 1 g of fresh plant material homogenized in Bead Ruptor Elite homogenizer (Omni International, Kennesaw, GA, USA) (remaining biomass was dried at 60 °C for 3 h, then for 1 h at 105 °C until a constant weight was attained) in a hand ceramic mortar with 2 × 10 mL of 100% ethanol (gradient grade ≥99.9%). Samples were prepared in triplicate, and concentrations were calculated per unit of sample dry weight. Next, the samples were allowed to stand for 24 h, then were filtered through a filter paper (Whatman, Grade 1, 11 µm) and centrifuged (Sigma 3–18 K, Sigma, Osterode am Harz, Deutschland) at 10,000 rpm for 7 min at 4 °C. A total of 50 μL of the sample was evaporated at 40 °C under vacuum (Heto VR-1, Heto Lab, Ikast, Denmark) collected, and diluted with 0.01% formic acid in methanol in a ratio of 1:10 and injected (2 μL) into the LC system consisting of Waters nanoACQUITY UPLC (ACQUITY UPLC I-Class, Waters, Milford, MA, USA) and nanoACQUITY HSS T3 column (phase C18, inner diameter 1 mm, length 50 mm, particle size 1.8 µm). The temperature of the column was 60 °C. The analytes were eluted at a flow rate of 0.15 mL/min with a linear gradient of 0.1% formic acid in water (A) and 0.1% formic acid in acetonitrile (B) as follows: 20% B for 1 min, from 20% to 40%; B in 9 min, from 40% to 49%; B in 12 min, from 49% to 95%; B in 13 min, 95%; B in 13 min, from 95% to 20%; B in 20 min. The conditioning time was 6 min.

Eluates were analyzed using a quadrupole time-of-flight mass spectrometer (Xevo G2 QTOF MS, Waters, Milford, MA, USA) equipped with an electrospray ionization source (ESI). A voltage of 0.5 kV was applied to the electrospray capillary. The source temperature was 120 °C, and the desolvation temperature was 350 °C. Nitrogen was used as a desolvation gas (800 L/h) and as a conical gas (45 L/h). The total ion current was recorded in positive ionization mode, with a scanning range of *m/z* 105 to 1100. Quantitative analysis was based on extracted ionic chromatograms for pseudomolecular ions *m/z*: DH 265.1440; H 263.1283; DHA 307.1545; HA 305.1389; DHM 333.1702; HM 331.1545; DHIB 335.1859; HIB/HMB/HIV 347.1859; HT 345.1702; and DHMB/DHIV 349.2015. The characteristic fragment ion for helenalin and its derivatives was *m/z* 245.1174, and that of dihydrohelenalin and derivatives was *m/z* 247.1324. Based on these ions, SLs were identified. The amounts of SLs were estimated by comparing the peak areas obtained for the particular SLs with the standard curve. Similarly to Spitaler et al., we also cannot separate isomers DHMB from DHIV and HMB from HIV with the analytical system used, so the content was given as a sum [34].

### 4.5. Sample Preparation for Histochemistry Analysis

Plant material, depending on the organ, were cut on a freezing Microtome Cryostat 1950 (Leica Micosystems, Nussloch, Germany) or, as was in the case of flowers, separated into fragments with a razor blade. Samples were prepared in a minimum of three replicates in different development stages. The obtained fragments were stained or treated with reagents directly on slides or watch glasses. The anatomical preparations were viewed and photographed using a Zeiss Axioscope with Microscope Camera Control Console MC 80 DX (Carl Zeiss, Oberkochen, Germany). The divided parts of the fresh flowers were viewed under polarized light, and their nature was confirmed by staining with Schiff’s reagent, resorcinol in HCl, and thymol-sulfuric acid reagents [86].

### 4.6. Immunolocalization of Farnesyl Policlonal Antibody

The immunolocalization procedure was based on the methods used by [87] with some modifications to the studied plant material. Farnesyl polyclonal antibody (PA1-12554, Thermo Fisher, Waltham, MA, USA) in ICC/IF was used to study the presence of farnesyl in the selected tissues of *Arnica montana* plants. Thin (40–50 µm) cross-sections of stems/leaves were cut from fresh material using a Microtome Cryostat 1950 (Leica Micosystems, Nussloch, Germany). Cells were fixed for 1 h in 4% (*w*/*v*) paraformaldehyde. After washing the samples with PBS (5 × 3 min), a polyclonal antibody was applied (diluted 1:10 in PBS) and incubated overnight at 4 °C in a humid chamber. The samples were washed with PBS (5 × 3 min), and the secondary antibody (Alexa Fluor TM 488 goat anti-rabbit IgG (H + L) Thermo Fisher) (diluted 1:100 in PBS) was applied. The samples were incubated overnight at 4 °C in the dark. The samples were washed with PBS (5 × 3 min each) and mounted on a mixture of PBS and glycerin. Observations were performed using a Leica epifluorescence microscope with a blue light excitation filter (470–490 nm). The following control probes were used: 1. Autofluorescence of the sample; 2. Control: secondary antibody only; 3. Control: primary polyclonal antibody only. Images were captured using a microscope equipped with an epifluorescence Zeiss Axioscope and 400 ISO film.

### 4.7. Histochemistry

The prepared fresh flower fragments were treated with a series of reagents to determine the location of the enzymatic activity and the presence of polysaccharides, lipids, and SLs (Table 3).

## 5. Conclusions

This study compared the SL synthesis capacity in the flower heads of three *Arnica* taxa. The highest amount of lactones was shown in *A. montana* cv. Arbo, half as much was observed in the wild species *A. montana* from western Poland, and the lowest content was revealed in *A. chamissonis*. In flowers in the bud phase, lactones were either absent or present in small amounts. Examination of the phases of flower development revealed very significant differences in the proportions of SL ester profiles proceeding with maturation, accompanied by an increase in the content of the more reactive H with two alkylation centers, compared to DH having one such center. Our results facilitate the harvesting of flowers to obtain specific derivatives for pharmaceutical and other purposes.

In all taxa, the concentrations of these metabolites increased with flower head maturation, indicating a protective function rather than an involvement in pollination. Additionally, we discovered that the style and stigma, stamens, receptacles, and pedicels do not synthesize lactones, whereas involucral bracts can form these substances. The largest amount of lactones is produced in parts related to the ripening of the ovary and pappus calyx, and less is produced in the perianth (florets’ tabularized corolla and expanded part of corolla). Large amounts of SLs present in the ovary may provide subsequent protection to seeds after their release from the inflorescence. The biological functions of SLs are known [93]: as protection against diseases and pests, allelopathic reactions, signals for the mobilization of arbuscular mycorrhizal fungi, initiation of symbiosis, and in physiological roles.

Here, we detected the presence of SLs in the floret of the pappus calyx, which is in disagreement with the accepted opinion that only glandular trichomes are places of lactone production.

All of epidermal structures contain lipids in the form of droplets or larger deposits, similar to the whorls of pappus bristles. The obtained data allowed us to propose that fructans and lactones co-localize with each other, and the presence of fructose polymers may exert a protective effect against living cells owing to their toxic effects. Similar to the action of pectin, which protects living trichomes, the cell wall subdomain accumulates essential oils in *Cannabis* and surrounding individual lipid vesicles, as was discovered by [37].

In view of the results obtained and the available data, it can be assumed that SLs can accumulate not only in the heads of secretory glands but also in the symplasts and apoplasts of the pappus and trichome cells. Vacuoles containing inulin can accumulate secondary metabolites with SLs and modulate the hygroscopic capacity of the pappus and accumulate water in the trichomes.

## Figures and Tables

**Table 1:** Sesquiterpene lactones levels ± SD [mg/g dw] in three taxa of *Arnica*: *Arnica montana* L., *Arnica montana* cv. Arbo, and *Arnica chamissonis* Less at different stages of development.

	*A. montana* L.		*A. montana* cv. Arbo		*A. chamissonis* Less.	
	Buds	Full Flowering	Buds	Full Flowering	Buds	Full Flowering
DH	0.04 ± 0.02	0.09 ± 0.06	0.02 ± 0.01	0.04 ± 0.01	-	-
H	0.09 ± 0.04	0.27 ± 0.05	0.14 ± 0.03	0.35 ± 0.09	0.24 ± 0.03	0.13 ± 0.05
DHA	-	0.03 ± 0.02	0.23 ± 0.01	0.52 ± 0.11	-	-
HA	-	-	0.18 ± 0.03	0.54 ± 0.12	-	-
DHM	0.53 ± 0.17	0.94 ± 0.46	0.54 ± 0.07	0.73 ± 0.12	-	-
HM	0.19 ± 0.08	0.72 ± 0.21	0.56 ± 0.06	1.44 ± 0.23	-	-
DHIB	0.38 ± 0.04	1.48 ± 0.76	0.46 ± 0.40	1.43 ± 0.28	-	-
HIB	0.15 ± 0.06	0.97 ± 0.26	1.07 ± 0.23	4.06 ± 0.87	-	-
DHT	1.70 ± 0.20	2.28 ± 0.87	0.75 ± 0.17	1.13 ± 0.19	-	-
HT	0.82 ± 0.06	2.49 ± 0.75	1.09 ± 0.09	2.75 ± 0.38	0.02 ± 0.01	0.01 ± 0.01
DHMB/DHIV	0.59 ± 0.11	1.22 ± 0.52	1.23 ± 0.11	2.47 ± 0.45	-	-
HMB/HIV	0.33 ± 0.06	1.15 ± 0.32	3.41 ± 0.37	9.42 ± 1.45	-	-
Total H	1.58 ± 0.31	5.59 ± 1.55	6.45 ± 0.75	18.57 ± 3.13	0.26 ± 0.03	0.14 ± 0.06
Total DH	3.25 ± 0.53	6.03 ± 2.69	3.24 ± 0.12	6.32 ± 1.17	-	-
Total SL	4.84 ± 0.67	11.63 ± 1.69	9.69 ± 0.68	24.88 ± 4.28	0.26 ± 0.03	0.14 ± 0.06

Helenalin (H); dihydrohelenalin (DH); acetylhelenalin (HA); acetyldihydrohelenalin (DHA); methacryloylhelenalin (HM); methacryloyldihydrohelenalin (DHM); isobutyrylhelenalin (HIB); isobutyryldihydrohelenalin (DHIB); tigloylhelenalin (HT); tigloyldihydrohelenalin (DHT); 2-methylbutyrylhelenalin (HMB); 2-methylbutyryldihydrohelenalin (DHMB); isovalerylhelenalin (HIV); isovaleryldihydrohelenalin (DHIV). Measurement uncertainty U = 18.82; *n* = 3; - = below to the limit of detection (LOD).

**Table 2:** Comparison of non-glandular and glandular trichomes on the achene surface (3.2 cm^2^) of *Arnica montana* and *Arnica montana* cv. Arbo.

*Arnica montana*			*Arnica montana* cv. Arbo		
Non-grandural Trichomes	Grandural Trichomes	Diameter of Grandular Trichomes [µm]	Non-grandural Trichomes	Grandural Trichomes	Diameter of Grandular Trichomes [µm]
16.95 ± 1.54	15.55 ± 3.33	32.33 ± 3.06	22.96 ± 2.63	18.09 ± 3.57	34.48 ± 3.83

*n* = 20.

**Table 3:** Histochemical methods used in the identification of compounds.

Histochemical Tests	Color of Reaction Products	References
Legal’s reaction for methylene and methyl ketone.	Red to dark brown; concentration dependent after adding CH_3_COOH red	Positive for helenalin [88]
Zimmermann reaction for methyl ketone and aldehyde.	Reddish purple	Positive for helenalin [88]
Schiff’s reagent for aldehydes.	Red or magenta	Negative for helenalin [41,88,89]
NADI for cytochrome oxidase	Stains lipids blue; fat inclusions, red-purple; and terpenes and resins, indigo to violet, according the proportion of oil and resin acids	[90]
Succinate dehydrogenase with Neo-T	Light blue	[91]
Vanillin for lactones	Stains helenalin yellow; after heating, turns reddish orange	[43]
p-dimethylamino-benzaldehyde for lactones	Greyish yellow	[43]
EP reaction for azulene and proazulenes	Green, blue or dark blue	[92]
1% EDTA in water lactones	Greyish black	[42]
2,4-Dinitrophenylhydrazine test for aldehydes and ketones, Brady’s reagent	Stains aliphatic carbonyls yellow; red to orange for aromatic carbonyls Precipitation on slides coated with glycerol, visible after several days	[89]

## Data Availability

All the date are in manuscript and Supplementary Material.

## Supplementary Materials

The following supporting information can be downloaded at: https://www.mdpi.com/article/10.3390/molecules28114379/s1, Table S1. The total content of SLs (%) in flower heads of *Arnica montana* L. collected from natural conditions and controlled plantations in Europe [27,28,29,30,31,94,95,96,97,98]. Table S2. Sesquiterpene lactone levels ± SD (mg/g dw) in three taxa of *Arnica*: *Arnica montana* L., *Arnica montana* cv. Arbo, and *Arnica chamissonis* Less in leaves and floral heads in full flowering stage. Table S3. Spatial distribution of helenalin and 11α, 13-dihydrohelenalin derivatives ± SD (mg/g dw) between examined parts of flowers during budding phase in disc and ray florets of *Arnica montana* cv. Arbo. Table S4. Distribution of helenalin and 11α, 13-dihydrohelenalin derivatives ± SD (mg/g dw) between isolated parts of flowers at the begging of flowering in disc and ray flowers of *Arnica montana* cv. Arbo. Table S5. Spatial distribution of helenalin and 11α, 13-dihydrohelenalin derivatives ± SD (mg/g dw) between examined parts of flowers during full flowering phase in disc and ray florets of *Arnica montana* cv. Arbo. Table S6. Distribution of helenalin and 11α, 13-dihydrohelenalin derivatives ± SD (mg/g dw) between studied flower parts at the end of the flowering phase in two types, disc and ray florets, of *Arnica montana* cv. Arbo. Table S7. Content of total sesquiterpene lactones ± SD (mg/g dw) in disc florets and green parts of floral heads during the full flowering phase of *Arnica montana* cv. Arbo. Table S8. Content of total sesquiterpene lactones (mg/g dw) in ray florets during the full flowering phase of *Arnica montana* cv. Arbo.

## Sample Availability

Samples of the compounds are not available from the authors.

## Abbreviation

ABA	abscisic acid
C/EBPβ	CCAAT/enhancer binding protein
CV	coefficient of variation
DAMPs	damage-associated molecular patterns
DH	11α, 13-dihydrohelenalin
DHA	acetyldihydrohelenalin
DHIB	isobutyryldihydrohelenalin
DHIV	isovaleryldihydrohelenalin
DHM	methacryloyldihydrohelenalin
DHMB	2-methylbutyryldihydrohelenalin
DHT	tigloyldihydrohelenalin
DMAPP	dimethylallyl pyrophosphate
FDS	farnesyl diphosphate synthase
FPP	farnesyl pyrophosphate
GM-CSF	regulatory link between granulocyte macrophage-colony stimulating factor
H	helenalin
HA	helenalin acetate
HIB	isobutyrylhelenalin
HIV	isovalerylhelenalin
HM	methacryloylhelenalin
HMB	2-methylbutyrylhelenalin
HPLC	high-performance liquid chromatography
HT	tigloylhelenalin
LOD	limit of detection
LOQ	limit of quantification
MALDI-MS	matrix-assisted laser desorption/ionization-mass spectrometry imaging
MAMPs	microbe-associated molecular patterns
MEP	2-C-methyl-D-erythritol-4-phosphate
MVA	mevalonate
NF-κB	nuclear factor kappa-light-chain-enhancer
PAP	primary cause of lung disease
PPI	isopentenyl diphosphate
RSD	relative standard deviation
SD	standard deviation
SLs	sesquiterpene lactones
SNARE	soluble N-ethylmaleimide-sensitive-factor attachment protein receptor
STPS	sesquiterpene synthases
UHPLC-MS	ultra-high performance liquid chromatography-mass spectrometry
