# Supplementary material for: Localization of Sesquiterpene Lactones Biosynthesis in Flowers of Arnica Taxa
Source: Molecules. 2023 May 27;28(11):4379. doi: 10.3390/molecules28114379 (PMC10254538; doi:10.3390/molecules28114379)
Supplement: Supplementary file 1 [file molecules-28-04379-s001.zip › Table S1.pdf]

Table S1. The total content of SLs (%) in flower heads of *Arnica montana* L. collected from natural conditions and controlled plantations in Europe.

Total content of SLs [%]	Habitat	References
0.31-0.91	natural site	[94]
0.80	natural site	[95]
0.5-0.94	natural site, Spain	[27]
1.61-2.85	various sites in mountainous areas, Spain	[28]
0.40-1.55	meadows and heathlands, Germany	[31]
0.45-1.51	cultivation in the natural site near Trento, Italy	[96]
0.54-1.50	cultivation from seeds from natural mountain sites	[97]
0.85-1.28	cultivation from estern Poland	[30]
0.46-1.39	experimental field located on mountain Tara in Western Serbia	[29]
1.21-3.12	experimental field located on mountain region on west Serbia	[98]
