# Supplementary material for: Localization of Sesquiterpene Lactones Biosynthesis in Flowers of Arnica Taxa
Source: Molecules. 2023 May 27;28(11):4379. doi: 10.3390/molecules28114379 (PMC10254538; doi:10.3390/molecules28114379)
Supplement: Supplementary file 1 [file molecules-28-04379-s001.zip › Table S2.pdf]

Table S2. Sesquiterpene lactone levels ± SD (mg/g dw) in three taxa of *Arnica*: *Arnica montana* L. *Arnica montana* cv. Arbo and *Arnica chamissonis* Less in leaves and floral heads in full flowering stage.

	A. montana cv. Arbo		*A. montana* L.		*A. chamissonis* Less.	
	leaves	floral heads	leaves	floral heads	leaves	floral heads
**DH**	-	-	-	-	-	-
**H**	-	0.74	-	2.33	0.54	0.35
**DHA**	0.65	0.14	-	-	-	-
**HA**	-	0.15	-	-	-	-
**DHM**	4.53	0.87	0.72	0.13	-	-
**HM**	-	1.04	-	2.80	-	-
**DHIB**	0.54	2.08	-	0.96	-	-
**HIB**	-	6.42	-	2.62	-	-
**DHT**	2.17	1.31	0.13	2.90	-	-
**HT**	-	0.86	-	3.32	-	-
**DHMB/DHIV**	0.52	1.36	-	-	-	-
**HMB/HIV**	-	12.18	-	2.58	-	-
Total H	-	21.38	-	13.65	0.54	0.35
Total DH	8.40	5.77	0.85	4.00	-	-
**Total SL**	**8.40**	**27.15**	**0.85**	**17.64**	**0.54**	**0.35**

Helenalin (H); dihydrohelenalin (DH); acetylhelenalin (HA); acetyldihydrohelenalin (DHA); methacryloylhelenalin (HM); methacryloyldihydrohelenalin (DHM); isobutyrylhelenalin (HIB); isobutyryldihydrohelenalin (DHIB); tigloylhelenalin (HT); tigloyldihydrohelenalin (DHT); 2-methylbutyrylhelenalin (HMB); 2-methylbutyryldihydrohelenalin (DHMB); isovalerylhelenalin (HIV); isovaleryldihydrohelenalin (DHIV). Measurement uncertainty U = 18.82; n = 3; - = below to the limit of detection (LOD).
