# Supplementary material for: Localization of Sesquiterpene Lactones Biosynthesis in Flowers of Arnica Taxa
Source: Molecules. 2023 May 27;28(11):4379. doi: 10.3390/molecules28114379 (PMC10254538; doi:10.3390/molecules28114379)
Supplement: Supplementary file 1 [file molecules-28-04379-s001.zip › Table S3.pdf]

Table S3. Spatial distribution of helenalin and 11α, 13-dihydrohelenalin derivatives ± SD (mg/g dw) between examined parts of flowers during budding phase in disc and ray florets of *Arnica montana* cv. Arbo.

	disc flowers				ray flowers				green parts	
**SL**	floret upper parts	floret middle parts	floret lower parts	floret pappus calyx	floret upper parts	floret middle parts	floret lower parts	floret pappus calyx	receptacle and phyllary bracts	peduncle
**DH**	-	1.19 ± 0.06	-	-	0.71 ± 0.01	-	-	-	0.26 ± 0.01	0.27 ± 0.06
**H**	-	-	-	-	-	-	-	-	-	-
**DHA**	-	-	-	-	-	-	-	-	-	-
**HA**	-	-	-	-	-	-	-	-	-	-
**DHM**	-	-	-	-	-	-	-	-	0.15 ± 0.02	-
**HM**	-	-	-	-	-	-	-	-	0.05 ± 0.01	-
**DHIB**	-	-	-	-	-	-	-	-	0.02 ± 0.01	-
**HIB**	-	-	-	-	0.16 ± 0.06	-	-	-	-	-
**DHT**	-	-	-	-	-	-	-	-	0.23 ± 0.01	-
**HT**	-	-	-	-	-	-	-	-	0.23 ± 0.01	-
**DHMB/DHIV**	-	-	-	-	-	-	-	-	0.03 ± 0.01	-
**HMB/HIV**	-	0.54 ± 0.04	-	-	2.36 ± 0.08	-	-	-	0.33 ± 0.02	-
Total H	-	0.54 ± 0.04	-	-	2.51 ± 0.14	-	-	-	0.61 ± 0.04	-
Total DH	-	1.19 ± 0.06	-	-	0.71 ± 0.01	-	-	-	0.69 ± 0.06	0.27 ± 0.06
**Total SL**	**-**	**1.73** ± 0.10	**-**	**-**	**3.23** ± 0.15	**-**	**-**	**-**	**1.30** ± 0.10	**0.27** ± 0.06

Helenalin (H); dihydrohelenalin (DH); acetylhelenalin (HA); acetyldihydrohelenalin (DHA); methacryloylhelenalin (HM); methacryloyldihydrohelenalin (DHM); isobutyrylhelenalin (HIB); isobutyryldihydrohelenalin (DHIB); tigloylhelenalin (HT); tigloyldihydrohelenalin (DHT); 2-methylbutyrylhelenalin (HMB); 2-methylbutyryldihydrohelenalin (DHMB); isovalerylhelenalin (HIV); isovaleryldihydrohelenalin (DHIV). Measurement uncertainty U = 18.82; n = 3; - = below to the limit of detection (LOD).
