# Supplementary material for: Localization of Sesquiterpene Lactones Biosynthesis in Flowers of Arnica Taxa
Source: Molecules. 2023 May 27;28(11):4379. doi: 10.3390/molecules28114379 (PMC10254538; doi:10.3390/molecules28114379)
Supplement: Supplementary file 1 [file molecules-28-04379-s001.zip › Table S4.pdf]

Table S4. Distribution of helenalin and 11α, 13-dihydrohelenalin derivatives (mg/g dw) between isolated parts of flowers at the begging of flowering in disc and ray flowers of *Arnica montana* cv. Arbo.

	disc flowers				ray flowers				green parts	
**SL**	floret upper parts	floret middle parts	floret lower parts	floret pappus calyx	floret upper parts	floret middle parts	floret lower parts	floret pappus calyx	receptacle and phyllary bracts	peduncle
**DH**	0.09 ± 0.00	0.01 ± 0.01	0.12 ± 0.02	0.06 ± 0.00	0.12 ± 0.02	-	-	-	0.02 ± 0.00	0.04 ± 0.00
**H**	-	-	0.16 ± 0.00	0.03 ± 0.00	-	-	-	-	-	-
**DHA**	-	-	-	-	-	-	-	-	-	-
**HA**	-	-	-	-	-	-	-	-	-	-
**DHM**	-	-	-	-	-	-	-	-	-	-
**HM**	-	-	0.54 ± 0.02	0.12 ± 0.00	-	-	0.34 ± 0.01	-	0.08 ± 0.00	-
**DHIB**	-	-	0.09 ± 0.02	0.01 ± 0.00	-	-	-	-	-	-
**HIB**	-	0.26 ± 0.02	1.15 ± 0.01	0.71 ± 0.01	0.06 ± 0.01	0.13 ± 0.02	0.67 ± 0.02	0.46 ± 0.11	0.05 ± 0.00	-
**DHT**	-	-	-	-	-	-	-	-	-	-
**HT**	-	-	0.48 ± 0.00	0.12 ± 0.00	-	-	0.26 ± 0.02	-	0.11 ± 0.01	-
**DHMB/DHIV**	-	-	-	-	-	-	-	-	-	-
**HMB/HIV**	0.09 ± 0.00	0.55 ± 0.02	2.36 ± 0.06	1.69 ± 0.01	0.27 ± 0.02	0.59 ± 0.02	1.95 ± 0.02	2.12 ± 0.24	0.21 ± 0.00	-
Total H	0.09 ± 0.00	0.80 ± 0.04	4.69 ± 0.04	2.67 ± 0.02	0.33 ± 0.03	0.72 ± 0.04	3.22 ± 0.07	2.58 ± 0.24	0.44 ± 0.01	-
Total DH	0.09 ± 0.00	0.01 ± 0.01	0.21 ± 0.04	0.08 ± 0.00	0.12 ± 0.02	-	-	-	0.03 ± 0.00	0.04 ± 0.00
**Total SL**	**0.18** ± 0.00	**0.81** ± 0.05	**4.89** ± 0.08	**2.75** ± 0.02	**0.46** ± 0.05	**0.72** ± 0.04	**3.22** ± 0.07	**2.58** ± 0.11	**0.47** ± 0.01	**0.04** ± 0.00

Helenalin (H); dihydrohelenalin (DH); acetylhelenalin (HA); acetyldihydrohelenalin (DHA); methacryloylhelenalin (HM); methacryloyldihydrohelenalin (DHM); isobutyrylhelenalin (HIB); isobutyryldihydrohelenalin (DHIB); tigloylhelenalin (HT); tigloyldihydrohelenalin (DHT); 2-methylbutyrylhelenalin (HMB); 2-methylbutyryldihydrohelenalin (DHMB); isovalerylhelenalin (HIV); isovaleryldihydrohelenalin (DHIV). Measurement uncertainty U = 18.82; n = 3; - = below to the limit of detection (LOD).
