# Supplementary material for: Localization of Sesquiterpene Lactones Biosynthesis in Flowers of Arnica Taxa
Source: Molecules. 2023 May 27;28(11):4379. doi: 10.3390/molecules28114379 (PMC10254538; doi:10.3390/molecules28114379)
Supplement: Supplementary file 1 [file molecules-28-04379-s001.zip › Table S5.pdf]

Table S5. Spatial distribution of helenalin and 11α, 13-dihydrohelenalin derivatives ± SD (mg/g dw) between examined parts of flowers during full flowering phase in disc and ray florets of *Arnica montana* cv. Arbo.

	disc flowers				ray flowers				green parts	
**SL**	floret upper parts	floret middle parts	floret lower parts	floret pappus calyx	floret upper parts	floret middle parts	floret lower parts	floret pappus calyx	receptacle and phyllary bracts	peduncle
**DH**	0.02 ± 0.01	0.06 ± 0.02	0.55 ± 0.02	-	0.06 ± 0.01	-	0.11 ± 0.02	-	0.06 ± 0.00	0.07 ± 0.00
**H**	-	-	0.37 ± 0.02	-	-	-	0.07 ± 0.02	-	-	-
**DHA**	-	-	-	-	-	-	-	-	-	-
**HA**	-	-	-	-	-	-	-	-	-	-
**DHM**	-	-	-	-	-	-	-	-	0.01 ± 0.00	-
**HM**	-	-	0.85 ± 0.02	-	-	-	0.57 ± 0.06	-	0.12 ± 0.01	-
**DHIB**	-	-	0.16 ± 0.02	-	-	-	0.05 ± 0.01	-	0.01 ± 0.00	-
**HIB**	-	0.33 ± 0.02	1.82 ± 0.02	1.38 ± 0.01	0.07 ± 0.02	0.53 ± 0.01	1.30 ± 0.08	2.10 ± 0.11	0.09 ± 0.00	-
**DHT**	-	-	-	-	-	-	-	-	0.03 ± 0.00	-
**HT**	-	-	0.71 ± 0.04	-	-	-	0.60 ± 0.10	-	0.19 ± 0.00	-
**DHMB/DHIV**	-	-	-	-	-	-	-	-	-	-
**HMB/HIV**	0.00	0.65 ± 0.05	3.63 ± 0.06	2.79 ± 0.02	0.28 ± 0.00	1.46 ± 0.04	3.44 ± 0.21	4.98 ± 0.50	0.36 ± 0.02	-
Total H	0.06 ± 0.00	0.99 ± 0.07	7.39 ± 0.16	4.17 ± 0.03	0.34 ± 0.02	1.99 ± 0.05	5.97 ± 0.47	7.08 ± 0.61	0.76 ± 0.03	-
Total DH	0.02 ± 0.01	0.06 ± 0.02	0.71 ± 0.4	-	0.06 ± 0.01	-	0.16 ± 0.03	-	0.11 ± 0.00	0.07 ± 0.00
**Total SL**	**0.07** ± 0.01	**1.05** ± 0.09	**8.10** ± 0.20	**4.17** ± 0.03	**0.41** ± 0.03	**1.99** ± 0.05	**6.13** ± 0.50	**7.08** ± 0.61	**0.87** ± 0.03	**0.07** ± 0.00

Helenalin (H); dihydrohelenalin (DH); acetylhelenalin (HA); acetyldihydrohelenalin (DHA); methacryloylhelenalin (HM); methacryloyldihydrohelenalin (DHM); isobutyrylhelenalin (HIB); isobutyryldihydrohelenalin (DHIB); tigloylhelenalin (HT); tigloyldihydrohelenalin (DHT); 2-methylbutyrylhelenalin (HMB); 2-methylbutyryldihydrohelenalin (DHMB); isovalerylhelenalin (HIV); isovaleryldihydrohelenalin (DHIV). Measurement uncertainty U = 18.82; n = 3; - = below to the limit of detection (LOD).
