# Supplementary material for: Localization of Sesquiterpene Lactones Biosynthesis in Flowers of Arnica Taxa
Source: Molecules. 2023 May 27;28(11):4379. doi: 10.3390/molecules28114379 (PMC10254538; doi:10.3390/molecules28114379)
Supplement: Supplementary file 1 [file molecules-28-04379-s001.zip › Table S6.pdf]

Table S6. Distribution of helenalin and 11α, 13-dihydrohelenalin derivatives ± SD (mg/g dw) between studied flower parts at the end of the flowering phase in two types, disc and ray florets of *Arnica montana* cv. Arbo.

	disc flowers				ray flowers				green parts	
**SL**	floret upper parts	floret middle parts	floret lower parts	floret pappus calyx	floret upper parts	floret middle parts	floret lower parts	floret pappus calyx	receptacle and phyllary bracts	peduncle
**DH**	0.05 ± 0.02	-	0.10 ± 0.01	-	0.23 ± 0.10	0.01 ± 0.01	-	-	0.10 ± 0.01	0.08 ± 0.00
**H**	-	-	0.19 ± 0.00	-	-	-	-	-	-	-
**DHA**	-	-	-	-	-	-	-	-	-	-
**HA**	-	-	0.09 ± 0.02	-	-	-	-	-	-	-
**DHM**	-	-	-	-	-	-	-	-	-	-
**HM**	-	-	1.05 ± 0.05	-	0.52 ± 0.03	0.23 ± 0.02	0.47 ± 0.06	-	0.08 ± 0.01	-
**DHIB**	-	-	0.19 ± 0.01	-	-	-	-	-	-	-
**HIB**	-	0.45 ± 0.01	1.77 ± 0.03	2.16 ± 0.11	2.25 ± 0.18	0.86 ± 0.02	0.85 ± 0.01	2.16 ± 0.05	0.05 ± 0.01	-
**DHT**	-	-	-	-	-	-	-	-	0.02 ± 0.00	-
**HT**	-	-	1.69 ± 0.04	-	0.76 ± 0.10	0.15 ± 0.02	1.39 ± 0.03	0.03 ± 0.02	0.17 ± 0.01	-
**DHMB/DHIV**	-	-	0.07 ± 0.01	-	-	-	-	-	-	-
**HMB/HIV**	0.26 ± 0.02	0.98 ± 0.06	4.67 ± 0.01	4.15 ± 0.32	5.02 ± 0.02	1.53 ± 0.02	4.44 ± 0.11	5.79 ± 0.36	0.32 ± 0.01	-
Total H	0.26 ± 0.02	1.44 ± 0.07	9.46 ± 0.15	6.31 ± 0.43	8.55 ± 0.33	2.77 ± 0.08	7.15 ± 0.21	7.99 ± 0.43	0.61 ± 0.04	-
Total DH	0.05 ± 0.02	-	0.36 ± 0.03	-	0.23 ± 0.10	0.01 ± 0.01	-	-	0.13 ± 0.01	0.08 ± 0.00
**Total SL**	**0.30** ± 0.04	**1.44** ± 0.07	**9.82** ± 0.18	**6.31** ± 0.43	**8.78** ± 0.43	**2.78** ± 0.09	**7.15** ± 0.21	**7.99** ± 0.43	**0.74** ± 0.05	**0.08** ± 0.00

Helenalin (H); dihydrohelenalin (DH); acetylhelenalin (HA); acetyldihydrohelenalin (DHA); methacryloylhelenalin (HM); methacryloyldihydrohelenalin (DHM); isobutyrylhelenalin (HIB); isobutyryldihydrohelenalin (DHIB); tigloylhelenalin (HT); tigloyldihydrohelenalin (DHT); 2-methylbutyrylhelenalin (HMB); 2-methylbutyryldihydrohelenalin (DHMB); isovalerylhelenalin (HIV); isovaleryldihydrohelenalin (DHIV). Measurement uncertainty U = 18.82; n = 3; - = below to the limit of detection (LOD).
