# Supplementary material for: Localization of Sesquiterpene Lactones Biosynthesis in Flowers of Arnica Taxa
Source: Molecules. 2023 May 27;28(11):4379. doi: 10.3390/molecules28114379 (PMC10254538; doi:10.3390/molecules28114379)
Supplement: Supplementary file 1 [file molecules-28-04379-s001.zip › Table S7.pdf]

Table S7. Content of total sesquiterpene lactones ± SD (mg/ g dw) in disc florets and green parts of floral heads during the full flowering phase of *Arnica montana* cv. Arbo.

	disc flowers						green parts		
	petals	stamens	stigmas	floret middle parts	floret lower parts	floret pappus calyx	phyllary bracts	receptacle	peduncle
**DH**	0.21 ± 0.02	-	-	0.17 ± 0.03	-	-	0.49 ± 0.01	-	-
**H**	-	-	-	-	-	-	-	-	-
**DHA**	-	-	-	-	-	-	-	-	-
**HA**	-	-	-	-	-	-	-	-	-
**DHM**	-	-	-	-	-	-	-	-	-
**HM**	-	-	-	0.05 ± 0.00	0.83 ± 0.06	-	0.05 ± 0.02	-	-
**DHIB**	-	-	-	-	-	-	-	-	-
**HIB**	0.04 ± 0.05	-	-	1.05 ± 0.03	1.25 ± 0.03	1.08 ± 0.06	-	-	-
**DHT**	-	-	-	-	-	-	0.01 ± 0.02	-	-
**HT**	-	-	-	-	0.24 ± 0.02	-	0.30 ± 0.02	0.04 ± 0.00	-
**DHMB/DHIV**	-	-	-	-	-	-	-	-	-
**HMB/HIV**	0.31 ± 0.01	-	-	1.14 ± 0.03	2.04 ± 0.02	1.13 ± 0.02	0.82 ± 0.02	0.09 ± 0.01	-
Total H	0.35 ± 0.06	-	-	2.24 ± 0.06	4.36 ± 0.13	2.21 ± 0.08	1.17 ± 0.06	0.13 ± 0.01	-
Total DH	0.21 ± 0.02	-	-	0.17 ± 0.03	-	-	0.50 ± 0.03	-	-
**Total SL**	**0.56** ± 0.08	**-**	**-**	**2.41** ± 0.09	**4.36** ± 0.13	**2.21** ± 0.08	**1.67** ± 0.09	**0.13** ± 0.01	**-**

Helenalin (H); dihydrohelenalin (DH); acetylhelenalin (HA); acetyldihydrohelenalin (DHA); methacryloylhelenalin (HM); methacryloyldihydrohelenalin (DHM); isobutyrylhelenalin (HIB); isobutyryldihydrohelenalin (DHIB); tigloylhelenalin (HT); tigloyldihydrohelenalin (DHT); 2-methylbutyrylhelenalin (HMB); 2-methylbutyryldihydrohelenalin (DHMB); isovalerylhelenalin (HIV); isovaleryldihydrohelenalin (DHIV). Measurement uncertainty U = 18.82; n = 3; - = below to the limit of detection (LOD).
