# Supplementary material for: Localization of Sesquiterpene Lactones Biosynthesis in Flowers of Arnica Taxa
Source: Molecules. 2023 May 27;28(11):4379. doi: 10.3390/molecules28114379 (PMC10254538; doi:10.3390/molecules28114379)
Supplement: Supplementary file 1 [file molecules-28-04379-s001.zip › Table S8.pdf]

Table S8. Content of total sesquiterpene lactones ± SD (mg/g dw) in ray florets during the full flowering phase of *Arnica montana* cv. Arbo.

**SL**	petals	style	floret lower parts	floret middle parts	floret pappus calyx
**DH**	0.36 ± 0.01	-	0.41 ± 0.01	0.54 ± 0.04	-
**H**	-	-	-	-	-
**DHA**	-	-	-	-	-
**HA**	-	-	0.16 ± 0.02	-	-
**DHM**	-	-	-	-	-
**HM**	0.01 ± 0.00	-	1.17 ± 0.02	0.22 ± 0.01	0.01 ± 0.01
**DHIB**	-	-	-	-	-
**HIB**	0.30 ± 0.01	-	1.09 ± 0.05	0.63 ± 0.02	0.42 ± 0.04
**DHT**	-	-	-	-	-
**HT**	0.10 ± 0.01	-	2.44 ± 0.03	0.31 ± 0.01	0.07 ± 0.00
**DHMB/DHIV**	-	-	-	-	-
**HMB/HIV**	1.10 ± 0.01	-	5.28 ± 0.21	1.74 ± 0.01	1.25 ± 0.10
Total H	1.52 ± 0.03	-	10.15 ± 0.13	2.90 ± 0.05	1.76 ± 0.15
Total DH	0.36 ± 0.01	-	0.41 ± 0.01	0.54 ± 0.04	-
**Total SL**	**1.88** ± 0.04	**-**	**10.56** ± 0.14	**3.44** ± 0.09	**1.76** ± 0.15

Helenalin (H); dihydrohelenalin (DH); acetylhelenalin (HA); acetyldihydrohelenalin (DHA); methacryloylhelenalin (HM); methacryloyldihydrohelenalin (DHM); isobutyrylhelenalin (HIB); isobutyryldihydrohelenalin (DHIB); tigloylhelenalin (HT); tigloyldihydrohelenalin (DHT); 2-methylbutyrylhelenalin (HMB); 2-methylbutyryldihydrohelenalin (DHMB); isovalerylhelenalin (HIV); isovaleryldihydrohelenalin (DHIV). Measurement uncertainty U = 18.82; n = 3; - = below to the limit of detection (LOD).
