# Supplementary figures and images for: Localization of Sesquiterpene Lactones Biosynthesis in Flowers of Arnica Taxa
Source: Molecules. 2023 May 27;28(11):4379. doi: 10.3390/molecules28114379 (PMC10254538; doi:10.3390/molecules28114379)

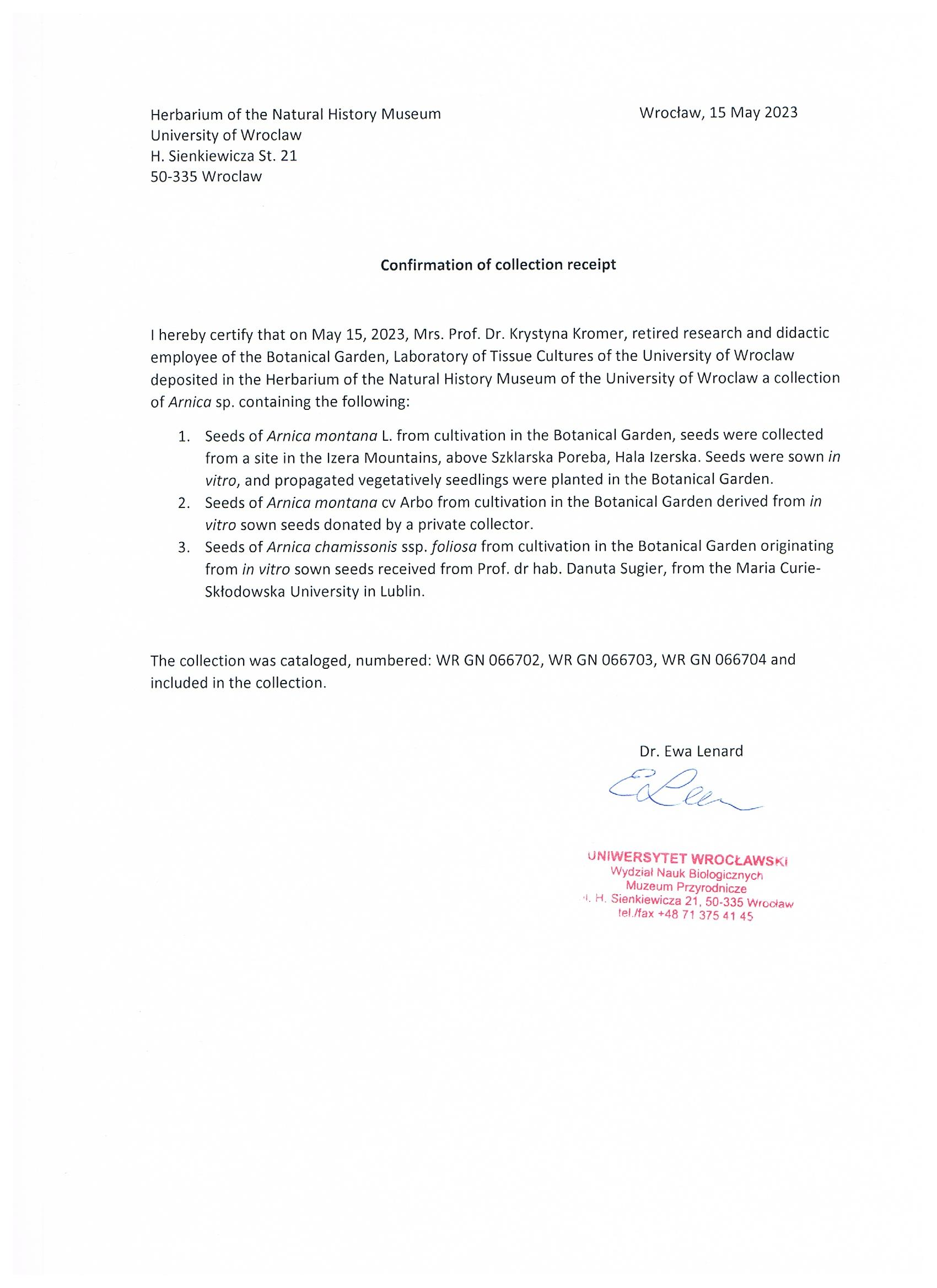

Supplement: Supplementary file 1 [file molecules-28-04379-s001.zip › voucher.tif]
